# *In vivo* and *in vitro* Characterization of a Partial Mu Opioid Receptor Agonist, NKTR-181, Supports Future Therapeutic Development

**DOI:** 10.3389/fpain.2021.695962

**Published:** 2021-08-23

**Authors:** Alex S. Lee, Suchi Tiwari, Isabel Bishop, Vartan Matossian, Nicole Romaneschi, Takahiro Miyazaki, Laurie VanderVeen, Jonathan Zalevsky, Kathryn DeFea, Catherine M. Cahill, Wendy M. Walwyn

**Affiliations:** ^1^Department of Psychiatry and Biobehavioral Sciences, University of California, Los Angeles, Los Angeles, CA, United States; ^2^Nektar Therapeutics, San Francisco, CA, United States; ^3^KiloDalton Consulting, Orinda, CA, United States; ^4^Division of Biomedical Sciences, School of Medicine, University of California, Riverside, Riverside, CA, United States; ^5^Brain Research Institute, University of California, Los Angeles, Los Angeles, CA, United States; ^6^Shirley & Stefen Hatos Center for Neuropharmacology, University of California, Los Angeles, Los Angeles, CA, United States; ^7^Jane & Terry Semel Institute for Neuroscience and Human Behavior, University of California, Los Angeles, Los Angeles, CA, United States

**Keywords:** NKTR-181, opioid use disorder, mu opioid receptor, analgesia, pre-clinical, abuse liability

## Abstract

Mu opioid receptor (MOPr) agonists are well-known and frequently used clinical analgesics but are also rewarding due to their highly addictive and often abusive properties. This may lead to opioid use disorder (OUD) a disorder that effects millions of people worldwide. Therefore, novel compounds are urgently needed to treat OUD. As opioids are effective analgesics and OUD often occurs in conjunction with chronic pain, these novel compounds may be opioids, but they must have a low abuse liability. This could be mediated by diminishing or slowing blood-brain barrier transport, slowing target receptor binding kinetics, and showing a long half-life. NKTR-181 is a PEGylated oxycodol and a MOPr agonist that has slowed blood-brain barrier transport, a long half-life, and diminished likeability in clinical trials. In this study, we examined the signaling and behavioral profile of NKTR-181 in comparison with oxycodone to determine whether further therapeutic development of this compound may be warranted. For this preclinical study, we used a number of *in vitro* and *in vivo* assays. The signaling profile of NKTR-181 was determined by the electrophysiological assessment of MOPr-Ca^2+^ channel inhibition in the nociceptive neurons of rodent dorsal root ganglia. Heterologous cell-based assays were used to assess biased agonism and receptor trafficking. Different rodent behavioral models were used to define the NKTR-181-induced relief of effective and reflexive nociception and drug-seeking behavior as assessed by an intravenous self-administration (IVSA) of NKTR-181. We found that NKTR-181 and oxycodone are partial agonists in G-protein signaling and Ca^2+^ channel inhibition assays and promote limited MOPr desensitization. However, NKTR-181 inhibits Ca^2+^ channels by a different mechanism than oxycodone and induces a different pattern of arrestin recruitment. In addition, NKTR-181 has a slower receptor on-rate and a slower rate of Ca^2+^ channel coupling than oxycodone. This signaling profile is coupled with a slower onset of antinociception and limited drug-seeking behavior in comparison with oxycodone. Together with its known long half-life and slow blood-brain barrier transport, these data suggest that NKTR-181 could be further studied as a pharmacotherapeutic treatment modality for OUD.

## Introduction

The prescription, diversion, and illicit use or production of opioid analgesics and opioids have emerged as a major societal concern fueling a concerted effort to identify novel treatments for chronic pain and the development of more effective treatments for those afflicted with substance use disorders. Opioid use disorder (OUD) affects over 27 million people worldwide (2016) and is considered as an epidemic in the USA as more than 100 people die daily due to opioid-related death. This disorder has become more prevalent during the COVID pandemic, with some opioid deaths thought to be from non-accidental from an intentional suicide (1–3). Current medications used for treating OUD include various formulations of the long-acting opioid agonists such as methadone and buprenorphine or the non-selective opioid antagonist naltrexone. However, each of these treatments has its limitations: methadone is only dispensed from licensed clinics and patients have to travel daily to get their dose. Buprenorphine is not orally bioavailable but formulated as a sublingual film that enters the blood stream directly through the mucosa under the tongue. Naltrexone generally shows the lowest compliance compared to the above noted agonists. Therefore, new treatment options are needed for those who are actively seeking treatments to manage their OUD.

In case of considering new treatment options for OUD patients, ideally such treatments should possess low abuse liability. One important factor in this regard is the rate at which a drug crosses the blood-brain barrier and enter the central nervous system (CNS). Opioids with a rapid entry into the CNS have a higher abuse liability than those that enter slowly (4). For example, the abuse of codeine is lower than that of morphine, heroin, or fentanyl because it must undergo first pass metabolism to generate its active metabolite to activate opioid receptors. An additional bonus of slowing CNS entry is that such drugs are less likely to cause life-threatening opioid-induced respiratory depression (5). One of the reasons for the success of methadone and buprenorphine as the treatment for OUD is their long half-lives. The premise of this beneficial pharmacokinetic property is that if the mu opioid receptor (MOPr) is occupied by these drugs, it is not available to be activated by short-acting, potent opioids such as morphine and fentanyl, thereby eliminating their ability to produce euphoria and other rewarding properties (6, 7). In addition, the pharmacological properties of a partial MOPr agonism produced by buprenorphine and a slower rate of agonist-receptor dissociation were correlated with reduced withdrawal and abuse liability (8).

NKTR-181 is a selective MOPr agonist consisting of a base morphinan pharmacophore, oxycodol, with an attached six-unit polyethylene glycol (PEG) chain that has been recently shown to produce antinociception in preclinical models (9). This opioid drug exhibits pharmacokinetic properties that make it a reasonable option for treating OUD. It has a slow onset of action with analgesic effects being only evident 1 h after oral or systemic (intraperitoneal) administration, the elimination half-life is 14 h and it has a slow entry into the CNS (10, 11). Indeed, in randomized double-blind human studies, NKTR-181 showed lower drug-liking compared to oxycodone in healthy subjects who reported the recreational use of opioids (12). Similarly, using the Misuse, Abuse, and Diversion Drug Event Reporting System (MADDERS®), Lanier et al. (13) identified the low rates of withdrawal and a low risk of abuse potential, diversion, or addiction associated with NKTR-181 in phase three trials of studying opioid-naïve and chronic non-cancer pain subjects.

Despite the preclinical data that show efficacy in various acute nociception assays, the effectiveness of NKTR-181 in measures of affective dimensions of persistent nociception has not been tested. Given that the bothersome emotional component of pain is more predictive of quality of life than the sensory pain experience, it is important to evaluate NKTR-181 in such assays. In this context, it is also important to understand if doses that produce antinociception elicit reinforcement in models of drug abuse. Moreover, it is unclear if NKTR-181 with its long half-life and reduced blood-brain barrier transport (10, 11) with a similar base structure as oxycodone, acts like oxycodone, a faster acting opioid that accumulates rapidly in the brain with a shorter half-life (14, 15). Such experiments would inform further development of NKTR-181 for the treatment of OUD. In this study, we characterized the signaling properties of NKTR-181 in a number of cell signaling assays, including in primary neuronal cultures. We also provide evidence that NKTR-181 alleviates the tonic aversive component of nociception at lower doses than that alleviating hypersensitivity associated with inflammation. Finally, we show that NKTR-181 had no effect in producing self-administration at greater doses (100-fold higher than oxycodone).

## Methods

### Rodents

All rodent experiments performed at University of California, Los Angeles (UCLA) were conducted in accordance with the AALAC Guide for the Care and Use of Laboratory Animals, were approved by the Office of Animal Research Oversight (OARO; protocol 1999; 179), and involved an initial power analysis to determine the sample size for *in vivo* and *in vitro* experiments involving animal tissues. This was further examined by an effect size analysis (G^*^power) of data captured from the initial cohorts of all behavioral groups and showed that power could be achieved with an *n* of 7–8. Self-administration studies were conducted at MPI Research, Inc. (Mattawan, MI, USA) in accordance with MPI Research Standard Operating Procedures. All experiments were in adherence with the ARRIVE guidelines.

#### *Exvivo* Primary Culture Studies

Four strains of mice were used; wild-type C57BL/6J mice (stock # 00664, Jackson Laboratories, Bar Harbor, ME, USA or the CSORDA animal breeding core at UCLA) and mice lacking both alleles of the following genes: (1) the MOPr (strain # 007559, Jackson Laboratories, Bar Harbor, ME, USA and bred by the CSORDA animal breeding core at UCLA), (2) βarr1 (stock # 01113, Jackson Laboratories, Bar Harbor, ME, USA), or (3) βarr2 (stock # 023852, Jackson Laboratories, Bar Harbor, ME, USA). All mice were on a C57BL/6J background, and the genetically engineered mice were backcrossed for at least 10 generations. Mice were housed in groups of two to four per cage on a 12-h reverse light/dark cycle, with food and water available *ad libitum*.

#### Nociceptive Behavior and Antinociception Profiling

All experiments were performed on 8–12-week-old male and female C57BL/6J mice (Jackson Laboratories, Bar Harbor, ME, USA). Mice were housed in groups of three to four per cage on a 12-h reverse light/dark cycle, with food and water available *ad libitum*. Mice were allowed to habituate to their housing environments for 1 week before handling, and the assignments of treatment were made prior to handling or experimentation. All mice in the same cage received the same treatment and pain condition. Experiments were conducted in the dark phase between 10:00 and 15:00 h. Experimenters handling mice were blind to carrageenan injection, sex, and drug treatment. Additionally, experimenters blind to experimental conditions analyzed the data sets. Mice were assigned to the conditions in a randomized block design so that the running of subjects counterbalanced factors such as time of day over experimental conditions. Mice were housed in groups of three to four per cage and were assigned treatments prior to any handling or experimentation. Experiments were conducted in successive replications, which were balanced with respect to experimental groups. All replications were balanced with respect to experimental groups. All behavior were performed in the dark (active) phase using a reverse light-dark cycle. Importantly, all tests were carried out by experimenters who were blind to experimental conditions. Additionally, all experiments were performed in low light conditions (LUX 5) depending on the experiment. Paw threshold testing was at the higher lux light level (LUX 10) while conditioned place preference (CPP)/aversion (CPA) experiments were conducted under the lowest light conditions. Mice were assigned to experimental conditions in a randomized block design so that factors such as the time of day were counterbalanced over the experimental conditions. For experiments on large numbers of groups, we completed them using successive cohorts, ensuring that experiments were carried out for all conditions within each cohort. All replications were balanced with respect to experimental groups.

#### Intravenous Self-Administration Studies

Experiments were conducted on male and female Sprague–Dawley rats (7.5 weeks old, Charles River, Portage, MI, USA) during the light cycle at MPI Research, Inc., Mattawan, MI, USA. Animals were then individually housed and maintained on a 12-h light/dark cycle with *ad libitum* food and water.

### Compounds

[D-Ala^2^, N-MePhe^4^, Gly-ol]-enkephalin (DAMGO) and fentanyl were obtained from Sigma, St. Louis, MO, USA. Morphine and oxycodone were obtained from the NIDA Drug Supply Program. NKTR-181 was supplied by Nektar Therapeutics (San Francisco, CA, USA). For cellular experiments, all compounds were made up as stock solutions in water and diluted and used on the day of the experiment. The same stocks were used for all cellular experiments. For behavioral outcomes, oxycodone and NKTR-181 were dissolved in sterile saline immediately prior to use. The protocols for the use and disposal of Schedule II compounds followed the guidelines outlined by the UCLA Department of Environmental Health and Safety.

### Cellular Experiments

#### Cultures

##### Dorsal Root Ganglia

Dorsal root ganglia (DRG) from both sides of the lumbar region (L1-6) were removed from male or female adult mice (2–5 months old) for all experiments except those assessing the effect of nociception, in which lumbar DRGs ipsilateral to the side of nociception were used. DRGs were collected in ice cold complete saline solution (CSS; in mM, NaCl 137, KCl 5.3, MgCl_2_ 1, Sorbitol 25, HEPES 10, and CaCl_2_: 3) and then dissociated in a series of collagenases; 1.25 U of TH (Sigma, St. Louis, MO, USA) with 250 nM EDTA for 20 min at 32°C in CO_2_ equilibrated CSS, transferred to fresh CSS containing 1.25 U of TM (Sigma, St. Louis, MO, USA) with 250 nM EDTA and 0.25 U papain (Sigma, St. Louis, MO, USA) and incubated for 10 min at 32°C, triturated through a series of fire-polished pasteur pipettes, spun (700 rpm, 3 min), and plated on poly-D-lysine (PDL ~125,000 kD, 1.25 μg/ml; Sigma, St. Louis, MO, USA) and the laminin (0.125 μg/ul; Invitrogen, Thermo Fisher, Waltham, MA, USA) coated 10 mm coverslip insert of MatTek dishes (MatTek Corporation, Ashland, MA, USA). They were incubated in B27 media containing neurobasal, Glumax, antibiotic-antimycotic (Life Technologies, Thermo Fischer Scientific, Carlsbad, CA, USA) supplemented with 10 ng/ml nerve growth factor (NGF) (Life Technologies, Carlsbad, CA, USA) as previously described (16).

##### HEK293 Cells

HEK293 cells, untransfected or transfected with fluorogen-activated peptide- (FAP-) tagged MOPr (Spectragenetics, Pittsburgh, PA, USA) were cultured in Dulbecco's Modified Eagle Medium (Gibco-Life Technologies, Thermo Fisher Scientific, Waltham, MA) supplemented with 10% fetal bovine serum (FBS, Gemini Bioproducts, Sacramento, CA, USA), 1% 100X Gibco® GlutaMAX™ supplement (Thermo Fischer, Life Technologies, Waltham, MA, USA), 0.5% 100X antibiotic-antimycotic (Thermo Fischer, Life Technologies, Waltham, MA, USA). Cells are grown at 37°C and 5% CO_2_.

#### Electrophysiology

**Figure 1:**
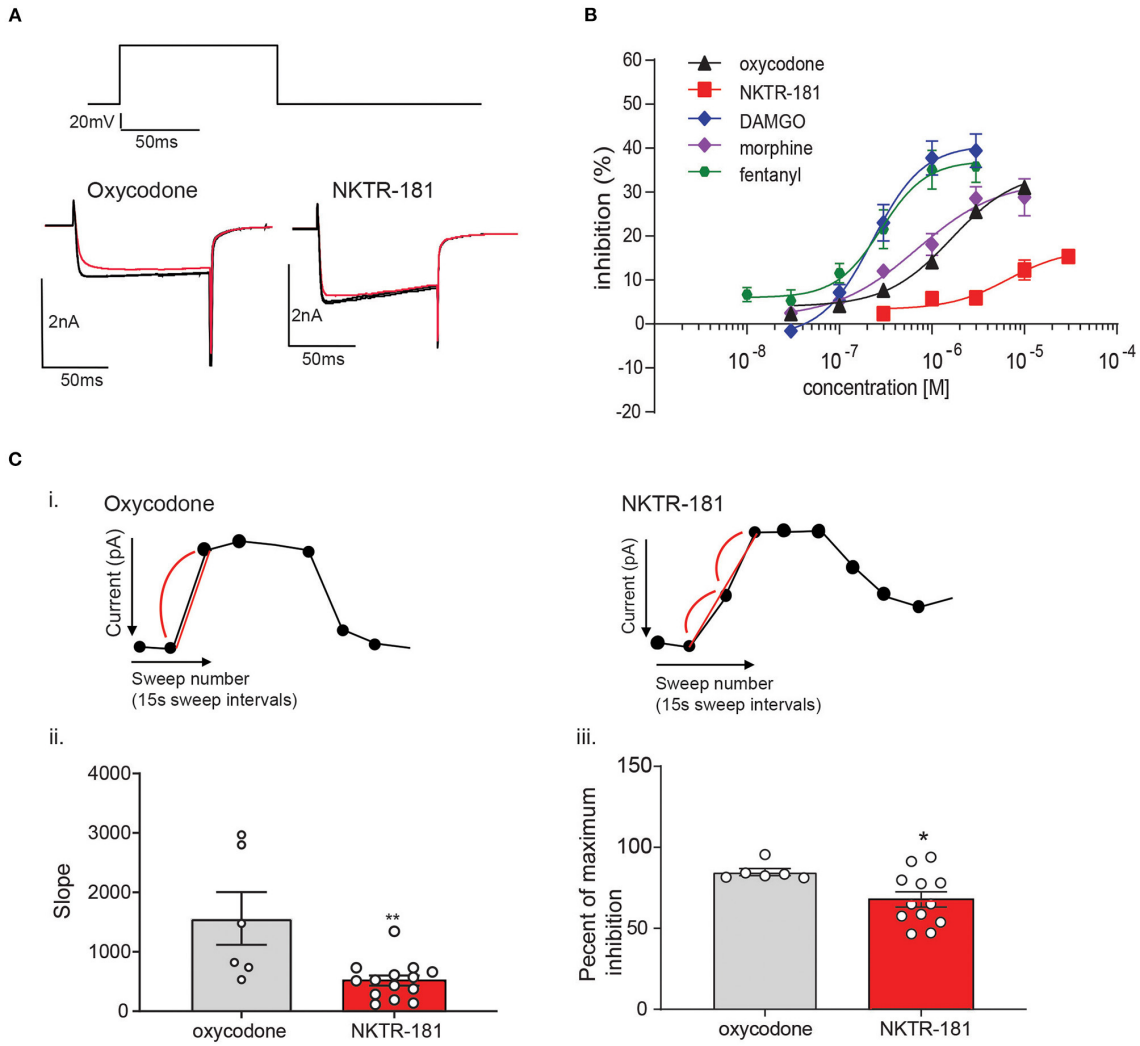
The profile of NKTR-181 inhibition of voltage-activated calcium channel currents (VACCs) in dorsal root ganglia (DRG) neurons. **(A)** Inhibition of VACCs was measured in dissociated and cultured adult DRG neurons. The current was obtained by a step voltage protocol from the holding voltage of −70 mV to +10 mV to open the Ca^2+^ channels, as shown in the top panel. This step protocol is repeated every 15 s to obtain a baseline Ca^2+^ current amplitude, after which mu opioid receptor (MOPr) agonist such as oxycodone or NKTR-181 is applied (shown in red) to inhibit the VACCs, and the agonist is then washed off. **(B)** In comparison with known MOPr agonists, NKTR-181 is both less efficacious and potent in this assay (Table 1). **(C)** Rate of inhibition. [C(i)] These exemplar recordings of the change in amplitude across time (each dot represents a 15 s sweep interval) induced by oxycodone (10 μM) or NKTR-181 (30 μM) were analyzed to obtain the rate of inhibition and the percentage inhibition obtained during the first sweep interval. NKTR-181 inhibited VACCs more slowly than oxycodone [C(ii)]; **p < 0.01 vs. oxycodone and obtained less percentage inhibition over time [C(iii)]; *p < 0.05 vs. oxycodone. Data are shown as mean ± SEM. Refer to Table 1 and Supplementary Table 2 for statistics.

After 24–36 h in culture, small to medium DRG neurons (capacitance <15 pF) were used for whole-cell patch-clamp techniques to record voltage-activated calcium channel currents (VACCs). The external solution contained (in mM): 130 TEA-Cl, 10 CaCl_2_, 5 HEPES, 25 D-glucose, and 0.25 tetrodotoxin (pH 7.2). Recording electrodes contained (in mM): 105 CsCl, 40 HEPES, 5 D-glucose, 2.5 MgCl_2_, 10 EGTA, 2 Mg^2+^-ATP, and 0.5 Na^+^-GTP (pH 7.2). VACCs were activated by an episodic protocol, which increased the holding voltage from −70 to 10 mv, and then keeping the voltage at 10 mv for 100 ms and returned it to −70 mv (Figure 1A). This induced a Ca^2+^ current with a maximum amplitude 5–10 ms after the initiation of the protocol, examples of which are shown in Figure 1A. Following a stable baseline obtained in the absence of an agonist, the agonist was applied at a flow rate of ~10 ml/min to assess the effect of this agonist for a minimum of two sweeps and then washed off. Each agonist was diluted in the external solution on the day of the experiment. In the experiments designed to assess the voltage-dependent component of agonist inhibition, the agonist was applied and after two sweeps, a pre-pulse protocol from −70 to +80 mV was either not applied or was applied before the standard depolarization protocol to evoke VACCs. An Axopatch 200B amplifier (Axon Instruments Inc., Foster City, CA, USA), a NIDAQ digitizer (NI USB-6221, National Instruments, Austin, TX, USA) and the winWCP software (U. Strathclyde, Glasgow, Scotland, UK) were used for these recordings to correct capacitance and series resistance and to compensate series resistance by 80–90% with the inclusion of a 10 μs lag. Leak currents were subtracted using a P/6 protocol. The recorded signals were acquired and analyzed using the winWCP (U. Strathclyde, Glasgow, Scotland, UK) and pCLAMP10 (Axon instruments) software.

**Table 1:** The dose-response curve of voltage-activated calcium channel currents (VACC) inhibition by NKTR-181 and other known Mu opioid receptor (MOPr) agonists.

**Compound**	**IC50 (nM)**	**Emax (% inhibition)**	**95% CI: span (% inhibition)**	***R* ^**2**^**
NKTR-181	6,659	17 ± 4	2–25	0.56
Oxycodone	1,697	34 ± 10	4–58	0.50
DAMGO	228	41 ± 4	23–64	0.72
Fentanyl	282	37 ± 4	20–43	0.67
Morphine	688	33 ± 6	14–49	0.65

*These parameters were obtained by semi-log dose-response curve-fitting and show NKTR-181 to be less potent and efficacious than other MOPr agonists*.

#### Bioluminescence Resonance Energy Transfer (BRET)

HEK293 cells were transfected 12–24 h after plating using FuGENE 6 reagent (1:3 ratio of DNA:Fugene 6, Promega, Madison, WI, USA). The DNAs used were β-arrestin-1-Renilla Luciferase8, β-arrestin-2-Renilla Luciferase8, mMOR-Venus, Gαi-Renilla Luciferase8, and Gβγ (a fusion of β and γ subunits). For acceptor donor titrations, β-arrestin-luciferase levels were held constant at 5 μg and mMOR-Venus varied from 2 to 15 μg. A ratio of 2.5:1 was used in all dose-response curves for β-arrestin-1 and β-arrestin-2 (Supplementary Figure 1, Supplementary Table 1). For G-protein activation, either MOR-Venus was co-transfected with Gαi-Luciferase and untagged Gβγ subunits. After 24 h, the cells were harvested and plated in white-bottomed 96-well microplates (Nunc™ F96 MicroWell™, Thermo Fisher Scientific, Waltham, MA, USA), previously coated with PDL (0.4 μg/ml; Sigma, St. Louis, MO, USA) and laminin (1 μg/ml; Sigma, St. Louis, MO, USA). As cells transfected with multiple constructs grew more slowly than singly transfected cells, we plated the cells co-transfected with multiple constructs at a density of 10,000 cells/well, whereas those transfected with a single construct were plated at a lower density of 5,000 cells/well. This resulted in the similar luminescence of ~10^6^ counts/well. After 24 h, cells were incubated with 9 μM coelenterazine h (50 μl reaction volume, Nanolight Technology, Pinetop, AZ, USA) for 5′, and diluted to the final concentration of 4.5 μM, addition agonist (final concentrations: 0 nM−100 μM) and the plate was immediately loaded into the GloMax® Discover Multimode Microplate Reader (Promega, Madison, WI, USA), equipped with a 482/35 nm single-band bandpass filter for measuring the emission in the donor channel and a 534/30 single-band bandpass filter for measuring the emission in the acceptor channel (BrightLine® from Semrock, Inc., Rochester, NY, USA). Data were collected every 1.4′ for 15–30′, using an integration time of 0.3 s at 30°C. For Gα recruitment experiments, coelenterazine and agonists were added simultaneously, and measurements were begun immediately. Background BRET (defined as the ratio observed in cells transfected with luciferase only) was subtracted from all data sets to get net BRET values. The netBRET signal at time = 0 was set to zero, and data were graphed as netBret vs. time.

#### Internalization

Untransfected or stably expressing FAP-tagged MOPr HEK cells were split at a density of 5 × 10^4^ cells/ml and 100 μl (5 × 10^3^ cells) divided into 1.5 ml microcentrifuge tubes. MOPr agonists of different concentrations, or medium alone, were diluted in a medium containing 200 nM of the non-permeant fluorescent peptide βRED-np (100 μl, 2x concentration) and added to each tube. The open tubes were returned to the incubator for 60′, 30′, or 10′. Ice-cold DMEM (1 ml) was added to each tube and then centrifuged (3′, 1,000 RPM, 4°C). The tubes were immediately placed on ice, the supernatant was removed, and 200 μl of non-permeable βGREEN fluorescent peptide (100 nM) was added to each tube and incubated for 10′. 1 ml of ice-cold medium was added to each tube, centrifuged (3′, 1,000 RPM, 4°C, the supernatant was removed, and the pellets were resuspended in 2% FBS/phosphate-buffered saline (PBS: Thermo Fischer, Life Technologies, Waltham, MA, USA) and transferred to individual flow tubes. They were run on an Attune NxT Flow using Attune NxT Software Cytometer (Thermo Fisher Scientific, Waltham, MA, USA) and the data transferred to FCSExpress v6 (deNovo Software, Glendale, CA, USA).

### D. Behavioral Experiments

#### Carrageenan-Induced Hyperalgesia

Mice received an intra-plantar (i.pl.) subcutaneous injection (30 μl) into the plantar glabrous left hindpaw using a 27-gauge needle. Mice were randomly divided into two cohorts: vehicle or carrageenan (2.0% w/v).

##### Thermal Withdrawal Thresholds

The antinociceptive and anti-hyperalgesic effects of oxycodone and NKTR-181 were assessed using the Hargreaves apparatus (IITC Life Science, Woodland Hills, CA, USA) (17). Thermal withdrawal latencies were measured following the drug or vehicle injection for *n* = 8 mice/sex per treatment. The light intensity was adjusted to produce baseline threshold latencies of 8–10 s. A cutoff latency was set at 20 s. To determine the effectiveness of oxycodone and NKTR-181 to produce antinociception or anti-hyperalgesic effects, oxycodone (1–3 mg/kg, i.pl.) was injected 3 h after i.pl. vehicle or carrageenan, and thresholds were determined every 10 min for 1 h. To assess the effects of NKTR-181 (30–100 mg/kg, i.pl.), it was injected immediately after threshold latencies were tested at 2 h following the i.pl. administration of vehicle or carrageenan based on pilot studies showing a delayed analgesic onset. Following the administration of NKTR-181, thermal thresholds were assessed every 10 min for 1 h beginning 70 min after NKTR-181 administration. All raw data are presented and are not transformed.

#### Conditioned Place Preference/Conditioned Place Aversion

The place preference paradigm was conducted using an unbiased, counterbalanced, two-chamber apparatus. Each square-floored box (28 × 28 × 19 cm) was divided into two equal-sized conditioning chambers. The two conditioning chambers were distinguishable by visual (black and white stripes or circles) and tactile cues using two types of metal flooring (soft screen or wire mesh), and different cleaning solutions (Simple green and Windex) were used for the two sides. To counterbalance the groups before the conditioning sessions, mice were placed in a Conditioned Place Preference (CPP) apparatus and allowed free access to both chambers. The time spent in each chamber was recorded over 30 min using an IR CCD camera attached to a computer running behavioral tracking software (Noldus Ethovision, Leesberg, VA, USA). *For CPP*: The drug-paired chamber was assigned such that any innate bias for one chamber over the other was balanced among treatment groups. Conditioning sessions consisted of mice (*n* = 8–12 per group) receiving six conditioning sessions, with 3 days of drug and 3 days of vehicle (saline) conditioning that consisted of a confinement in the appropriate chamber for 30 min. For oxycodone conditioning, animals were placed in the conditioning chamber immediately after injection, whereas mice that received NKTR-181 were injected and placed in the home cage for 1 h prior to placing in the conditioning chamber for 30 min. The delay in timing for conditioning exposure environment for NKTR-181 was selected due to the delayed onset of drug effects in nociception assays. The CPP assay was conducted in pain-naïve animals to assess the potential rewarding effects of oxycodone and NKTR-181. On the post-conditioning day, animals were allowed free access to both chambers in a drug-free state, and the time spent in the drug-paired chamber was measured over 30 min. Data are presented as the CPP score (time in postcondition drug chamber—time in precondition drug chamber)—(time in post condition saline chamber—time in precondition saline chamber). *For Conditioned Place Aversion (CPA)*: As with the CPP paradigm, the carrageenan-paired chamber was assigned such that any innate bias for one chamber over the other was balanced. Conditioning sessions consisted of mice (*n* = 8 per group) receiving two conditioning sessions; the 1st day all mice received i.pl. saline and the 2nd day all mice received i.pl. carrageenan (2% w/v). The conditioning chamber was counterbalanced but mice always received the nociceptive stimulus on the 2nd day of conditioning. Conditioning commenced 1 h after i.pl. injections that consisted of a confinement in the appropriate chamber for 45 min. To assess the ability of oxycodone to prevent nociception-induced CPA, mice were injected with oxycodone 1 h following carrageenan injection and immediately placed in the conditioning chamber. For NKTR-181, mice were injected with the opioid and carrageenan at the same time and not placed in the conditioning chamber until 1 h after the injections. Thus, to determine the effectiveness of oxycodone or NKTR-181 to block carrageenan-induced CPA, oxycodone was injected immediately prior to conditioning, while NKTR-181 was injected at the same time as carrageenan due to its delayed onset of effects. Oxycodone and NKTR-181 were administered on only the carrageenan conditioning days and not on the saline days. In the post-conditioning day, animals were allowed free access to both chambers in a drug-free state, and the time spent in the drug-paired chamber was measured over 30 min. The CPA score was calculated in the same way as the CPP score noted above.

#### Intravenous Self-Administration (IVSA)

All animals were surgically implanted with chronic indwelling jugular catheters (MPI Research, Inc., Mattawan, MI, USA) per Charles River Laboratories standard operating procedures as discussed in previous publications (18). Animals were then individually housed and maintained on a 12-h light/dark cycle with *ad libitum* food and water. About 1 week following surgery, access to food was limited to maintain 85–95% of body weight, with supplemental food (Dustless Precision Pellets, 45 mg, Rodent Grain-Based Diet, Bio-Serv, Flemington, NJ, USA) earned during operant sessions. These sessions were 30 min long or to a maximum of 50 reinforcers, whichever came first. Once consistent responding was obtained at a fixed ratio of one (one lever press for each reinforcer, FR1) schedule, cocaine (0.56 mg/kg/infusion) was included as a reinforcer for one session simultaneously with the food reward to a maximum of 10 reinforcers. Thereafter, food rewards were discontinued as was food restriction, and the schedule increased to FR10 with stable responding, <20% variation in responding over 3 consecutive days, required before moving to the next schedule. The reinforcer was then changed to hydrocodone (0.18 mg/kg/infusion) under an FR10 schedule. When stable responding was obtained for hydrocodone, 46 males and 42 females underwent three sessions on 3 consecutive days receiving saline as the reinforcer. This was followed by a trial, consisting of three sessions, 60 min in length, and with no limit on the number of drug reinforcers obtained, over 3 consecutive days. This protocol was repeated for each dose of each drug with each trial interspersed by three sessions of hydrocodone to a maximum of 1 h or 10 reinforcers, whichever came sooner.

### Statistics and Data Analysis

#### Agonist Inhibition

Stable recordings were fitted by a linear function to compare, by extrapolation, control current amplitude with the current amplitude recorded in the presence of the opioid receptor agonist used. Each datapoint comprises a minimum of six recordings obtained from two to four mice. Details of the specific statistical analyses used for each experimental protocol (Student's *t*-test, one- or two-way ANOVA, or linear mixed model analysis with Tukey, Sidak's, or Dunnett's *post hoc* test; GraphPad Prizm v7 or 8) are provided in the statistics table (Supplementary Table 2).

#### BRET

Dose-response curves were fitted from graphs of percent maximum DAMGO response vs. Log [agonist] using a four-parameter, variable slope, least squares regression analysis (GraphPad Prizm v8). The same data were also fit with an operational least squares regression model for partial agonism, using DAMGO as the reference compound, to determine the transducer constant, $\sigma_{lig}=\;\log\frac{{\tau /K\;}_{lig}}{{\tau /K}_{ref}}$. Bias calculations were then calculated as follows: $bias\;\left(\beta \right)\;=\;\frac{\left(\sigma_{path1}-\sigma_{path2}\right)}{\sqrt[{2}]{2}}$ (19–21).

Bias can also be calculated using the equi-active model, which relies on the ratio of Emax to EC50 derived from the four-parameter linear regression dose curves and designating a reference compound (DAMGO) as having no bias.


$$\begin{array}{l} bias\left(\beta \right)=\log\left({\left(\frac{RA_{\text{path}1}}{RA_{\text{path}2}}\right)}_{\text{lig}}÷{\left(\frac{RA_{\text{path}1}}{RA_{\text{path}2}}\right)}_{\text{ref}}\right) \end{array}$$

where $RA=\frac{Emax_{\text{path}1}}{{EC50}_{\text{path}1}}\times \frac{{EC50}_{\text{path}2}}{Emax_{\text{path}2\;}}$ (equi-active model).

For kinetic analysis, the time to half-maximal response was determined by least squares regression analysis of percent maximum response vs. time graphs using a one-phase decay model. For acceptor/donor curves, data were fit by non-linear regression using a one-site binding model. Statistically significant differences in Emax, EC50, RA, τ/K, and rate constants are determined using ANOVA with Tukey post-tests and unpaired, two-tailed *t*-tests, and all statistics are shown in Supplementary Table 2.

#### Internalization

After setting up a template based on the fluorescence present in unlabeled, viable cells and in those labeled with either or both fluorescent labels, we quantified the geometric mean of the relative fluorescence intensity expressed as the percentage of maximum (βRed alone) and minimum (βRed and βGreen) possible values. Data were fitted to a sigmoidal dose-response curve for each timepoint (10′, 30′, and 60′) or analyzed by one-way ANOVA (GraphPad Prizm v8) with further details provided in Supplementary Table 2.

#### Desensitization

Desensitization was defined by two protocols; rapid and chronic desensitization. To assess rapid desensitization, adult DRG neurons were voltage-clamped, and a stable baseline was obtained. The target agonist was then perfused continuously over the cell for 2′ and the resultant inhibition assessed every 15 s, and washed off. Desensitization was expressed as the percent loss of inhibition between the first and the last sweep in the presence of an agonist. To assess a chronic desensitized adult, DRGs neurons were incubated with the target drug for 10, 30′, 120′, and 240′ at 37°C, 5% CO_2_. The drug was replaced by perfusion with ~30 ml of external solution prior to assessing the inhibition obtained by the full agonist, DAMGO (1 μM). All statistics are shown in Supplementary Table 2.

#### Nociception Behavior

Data are expressed as scatter plots with mean and SEM (GraphPad Prism v9). All behavioral data met the assumptions of a general linear model, were subjected to factorial ANOVAs, and were analyzed by repeated measures (Hargreaves test) mixed effect two-way ANOVAs followed by Sidak's multiple comparison tests. For CPP and CPA data, the CPP and CPA scores are presented, and data were analyzed by one sample *t*-test with a hypothetical value of zero. Further statistical details for all raw data files are provided in Supplementary Table 3.

#### IVSA

An *n* of six females and six males for each dose and drug (saline, oxycodone, and NKTR-181) was obtained and the data presented as the average number of reinforcers were obtained over the hour-long session for sessions two and three, with the first session being excluded due to a transition effect from the previous session. After removing outliers using the ROUT method, *Q* = 1, the data were analyzed by mixed-effects analysis with repeated measures to compare the effects of saline or drug on the number of reinforcers obtained to compare the effects of saline or drug on the number of reinforcers obtained (GraphPad Prism v9). The total amount of drug received during the hour session was also analyzed by one-way ANOVA with Tukey's *post hoc* test to compare the amount of drug received across the three doses used for each drug, and further details are provided in Supplementary Table 3.

## Results

### Cellular Experiments

#### NKTR-181 Inhibition of VACCs in Adult DRG Neurons Is Both Less Potent and Efficacious Than Oxycodone and Other Known Mu Agonists

Gβγ-mediated inhibition of VACCs by MOPr agonists in DRG neurons is a known physiological effect of these agonists (22) that, in binding to peripheral MOPrs, induces analgesia in the pain state (23). We used this physiologically relevant assay to compare and contrast the efficacy and potency of NKTR-181 in comparison with a panel of known full or partial agonists to understand where NKTR-181 and oxycodone fit within this panel. An example of this technique is shown in Figure 1A and B shows the compiled data. We found that, in comparison with the known full agonists, DAMGO, fentanyl, and the partial agonist, morphine, NKTR-181 showed both less efficacy and potency (Figure 1B, Table 1). Although oxycodone is considered a full agonist as an antinociceptive compound (24), in this assay the dose-response profile of oxycodone was similar to that of morphine, a known partial agonist (20).

#### NKTR-181-VACC Inhibition Is Slower Than Oxycodone-VACC Inhibition

We next assessed the rate of inhibition obtained by NKTR-181 in comparison with oxycodone using the highest concentration of the respective dose-response curve (Figure 1C; NKTR-181; 30 μM and oxycodone; 10 μM). The drug-induced change in current amplitude was fitted with a linear equation using the baseline amplitude before the agonist was applied and when the drug had reached peak inhibition, as shown in Figure 1C(i). An unpaired two-tailed *t*-test showed a slower rate of inhibition by NKTR-181 compared with oxycodone [Figure 1C(ii), Supplementary Table 2]. We also assessed the maximum inhibition obtained by one epoch, 15 ms, once the drug was applied, as shown in Figure 1C(i), and found that, within this timeframe, NKTR-181 induced less inhibition, expressed as a percent of the total inhibition than oxycodone [Figure 1C(iii), Supplementary Table 2]. Both data sets showed these samples to have a different variance (slope; *p* < 0.001 and percent inhibition; *p* < 0.05), which remained uncorrected in the analysis.

#### NKTR-181 Is a Partial Agonist at G-Protein and β-Arrestin Signaling (βarr)

**Figure 2:**
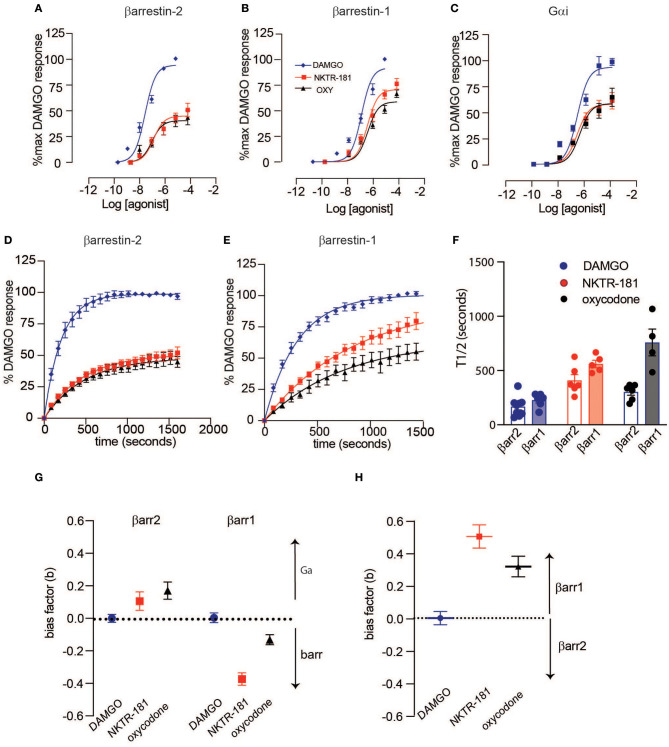
NKTR-181-induced G-protein and β-arrestin recruitment to MOPr. HEK293 cells, transfected with MOPr-venus and either GNAi1-luciferase, luciferase-βarr2, or luciferase-βarr1, were treated with [D-Ala^2^, N-MePhe^4^, Gly-ol]-enkephalin (DAMGO) (0–10 βM), NKTR-181 (0–100 βM), or oxycodone (0–100 βM). **(A–C)** Dose-response curves showing agonist-activated NetBRET values [E_535_/E_490_ minus the background signal observed with luciferase-tagged constructs alone and normalized to baseline bioluminescence resonance energy transfer (BRET) in untreated cells] as a percentage of the maximum DAMGO-induced response on the *y*-axis, and log agonist concentration on the *x*-axis for βarr2 **(A)**, βarr1 **(B)**, and Gαi **(C)**. Data were fitted with a four-parameter, variable slope model with Hill Slopes constrained to one. **(D–F)** Time course of βarr2 **(D)** and βarr1 **(E)** recruitment at DAMGO (10 μM), NKTR-181 (100 μM), and oxy (100 μM), fitted with a one-phase decay model, and recruitment rate comparison **(F)**. Bias factors were calculated for Gαi vs. β-arrestin **(G)** or βarr1 vs. βarr2 **(H)**. Refer to Supplementary Table 1, Tables 2, 3 for statistics. Refer to Supplementary Figure 2 for the same analysis with morphine and fentanyl.

Using BRET, with DAMGO as a reference compound, we observe that NKTR-181 and oxycodone behave similarly with respect to G-protein and β-arrestin recruitment: both are partial agonists, for the recruitment of Gαi, βarr1, and βarr2 to MOPr, but have a higher efficacy for βarr1 recruitment than for Gαi or βarr2 (Figures 2A–C, Table 2). Morphine is 6-fold more potent than NKTR-181 at Gαi recruitment, but has similar potency and efficacy at βarr2 recruitment and is less efficacious than either NKTR-181 or oxycodone at βarr-1 recruitment (Table 2, Supplementary Figure 2). Another consideration when evaluating partial agonists, beyond comparing EC50 and maximum potency, is the intrinsic efficacy of a compound, which takes into account the receptor occupancy required to achieve a half-maximal response (20). Competitive binding assays with radiolabeled naloxone show that NKTR-181 has a 5- and 10-fold lower affinity for MOPr than oxycodone and DAMGO, respectively. Binding kinetics suggest that the difference in NKTR-181 and oxycodone affinity for MOPr is due to a 10-fold slower on-rate for NKTR-181 (Supplementary Figures 3, 4). Fitting dose-response curves using an operational model for partial agonists (19), with DAMGO as the reference compound, reveals a small but statistically significant higher transducer constant (σ = △Logτ/K_A_) for NKTR-181-stimulated βarr1 recruitment to MOPr compared to oxycodone, suggesting that NKTR-181 may require a lower receptor occupancy to elicit βarr1 recruitment, despite the fact that the two agonists do not show a significant difference in efficacy and efficiency (Table 2). In addition to the maximal BRET signal, the kinetics of βarr recruitment to MOPr can reveal subtle differences in signaling. In general, the time it takes to reach maximum BRET signal is lower for a full agonist than a partial agonist. Both NKTR-181 and oxycodone promote a slower βarr1 and βarr2 recruitment than DAMGO or fentanyl but the rate for oxycodone-induced recruitment of βarr1 is significantly slower than the recruitment of βarr2 while NKTR-181 promotes the recruitment of both β-arrestins at approximately the same rate (Figures 2D–F, Supplementary Figures 2D–F). In contrast, NKTR-181 promotes the recruitment of βarr1 and βarr2 at approximately the same rate (Figures 2D–F). These differences in NKTR-181 and oxycodone recruitment of βarr1 are reflected when bias factors are determined. Using the operational model, we observe that both NKTR-181 and oxycodone have a small bias for βarr1 recruitment over Gβ recruitment, and NKTR-181 has a significantly higher β-arr1 bias than oxycodone. In contrast, NKTR-181 had a small bias toward Gαi over βarr2, but this was not statistically significant, while oxycodone had a statistically significant bias for Gαi over βarr2 (Figure 2G, Table 3). Both NKTR-181 and oxycodone also show a bias for the recruitment of βarr1 over βarr2 (Figure 2H, Table 2) and NKTR-181 once again shows a statistically significant higher βarr1 bias than oxycodone. In contrast, morphine appears to be biased toward Gαi recruitment over both βarr2 and βarr1 recruitment (Supplementary Figure 2, Table 3). These findings are corroborated by the bias factors obtained using the equi-active model, or by plotting Emax values for βarr1 and βarr2 recruitment against Gαi recruitment, although differences between oxycodone and NKTR-181 are only statistically significant using the operational model.

**Table 2:** Recruitment of Gαi and β-arrestins to MOPr.

**Recruitment**	**Agonist**	**Emax**	**_**Log**_EC50 (M)**	**σ**
Gα_i_	DAMGO	100 ± 5.3	−7.73 ± 0.13	0
	NKTR-181	61.84 ± 0.5^**^	−6.42 ± 0.19^*^	0.18 ± 0.07
	oxycodone	62.54 ± 0.9^**^	−6.45 ± 0.19^*^	0.006 ± 0.07
	morphine	795 ± 0.4	−7.18 ± 0.18^#^	0.17 ± 0.09
	fentanyl	93.35 ± 0.3^#^	−7.84 ± 0.13^#^	0.14 ± 0.1
β-arr2	DAMGO	100 ± 3.7	−7.540 ± 0.07	0
	NKTR-181	47.73 ± 0.3^*^	−6.840 ± 0.178^*^	−0.012 ± 0.04
	oxycodone	433 ± 0.5^*^	−7.00 ± 0.22	−0.17 ± 0.05
	morphine	39.52 ± 0.2^*^	−7.20 ± 0.15	−0.36 ± 0.03^*#^
	fentanyl	745 ± 0.8	−8.10 ± 0.22^*#^	0.21 ± 0.17^#^
β-arr1	DAMGO	100 ± 5.5	−6.940 ± 0.09	0
	NKTR-181	76.94 ± 0.6^*^	−6.450 ± 0.12^*^	0.33 ± 0.06^*^
	oxycodone	63.93 ± 0.8^**^	−6.410 ± 0.12^*^	0.07 ± 0.05^#^
	morphine	453 ± 0.6^**#^	−6.30 ± 0.18^*^	−0.45 ±0.06^*#^
	fentanyl	91.45 ± 0.3	−8.00 ± 0.12^**#^	0.24 ± 0.75^*^

*EC50 and Emax values were calculated using a nonlinear regression, four-parameter, variable slope model. Transduction coefficients were determined by using the operational partial agonist curve fit, with [D-Ala^2^, N-MePhe^4^, Gly-ol]-enkephalin (DAMGO) as the reference and KA values derived from 3H-Naloxone competitive binding experiments*.

^*^, ^**^ indicates statistically significant difference from DAMGO (p <0.05, − <0.001), respectively, and

# *indicates a statistically significant difference from NKTR-181 (p <0.05)*.

**Table 3:** G-protein vs. β-arrestin bias: Bias factors were determined using the operational model (based on the ratio of transduction constants σ for each pathway).

**Agonist**	**Gαi vs βarr2 βarr1**	**βarr1 vs βarr2**
**DAMGO**	**Unbiased**		**Unbiased**
NKTR-181	0.11 ± 0.06	−0.37 ± 0.04^*^	0.51 ± 0.07^*^
Oxycodone	0.17 ± 0.08^*^	−0.14 ± 0.07^*^^#^	0.32 ± 0.06^#^
Morphine	0.39 ± 0.07^*^^#^	0.41 ± 0.027^*^^#^	−0.05 ± 0.054^#^
fentanyl	−0.05 ± 0.14	−0.07 ± 0.05	0.023 ± 0.054^#^

^*^,^**^ Indicates a statistically significant difference compared to DAMGO, p < 0.05, p < 0.005, respectively;

# *indicates a statistically significant difference from NKTR-181, p <0.05; n = 7 for Gαi bias*.

#### NKTR-181 and Oxycodone Both Induce Minimal Desensitization in Adult DRG Neurons

**Figure 3:**
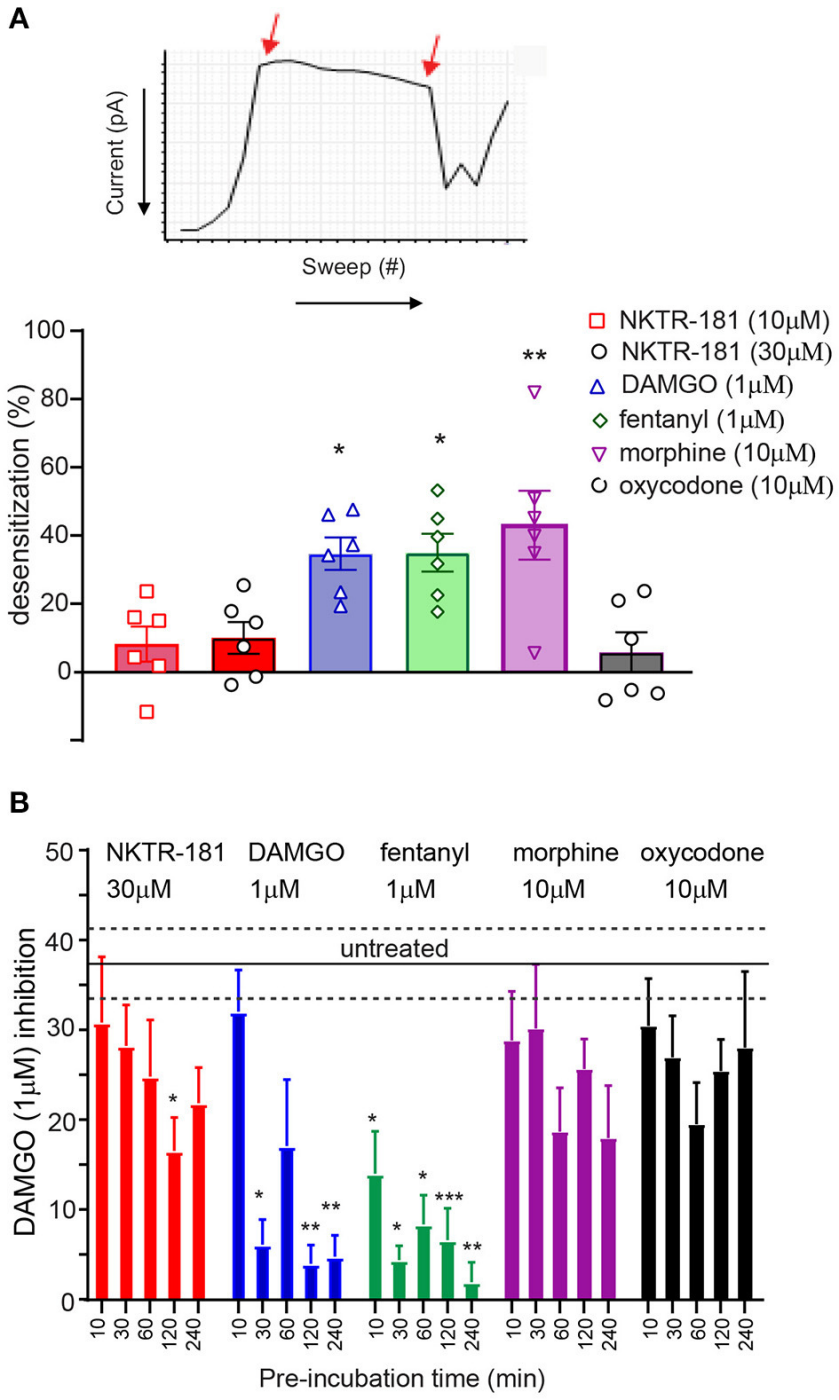
NKTR-181 results in minimal desensitization of MOPr-VACC inhibition. **(A)** Rapid desensitization. **(A)** VACC inhibition by mu agonists desensitizes over time, as shown by the decrease in current when the agonist is continuously perfused and the loss of inhibition assessed with each sweep, as shown by the two arrows 2′ apart. **(A)** These recordings were obtained following the application of each agonist and show that DAMGO, fentanyl, and morphine, but not NKTR-181 or oxycodone, induce desensitization; *, **, *p* < 0.05, and 0.01, respectively, vs. NKTR-181 30 μM. **(B)** *Chronic desensitization*: VACC inhibition by MOPr agonists also desensitizes the following periods of longer agonist exposure. In this assay, DRG neurons were pre-incubated with different agonists for increasing periods of time and doses. The agonists were then washed off and VACC inhibition assessed by 1 μM DAMGO to define MOPr-VACC inhibition compared with that of untreated cells. DAMGO and fentanyl desensitized MOPr-VACC inhibition over time. NKTR-181 induced limited desensitization after 2 h of pre-incubation and morphine and oxycodone resulted in no desensitization. *, **, and *** *p* < 0.05, 0.01 and 0.001 vs. untreated, respectively. Data are shown as mean ± SEM. Refer to Supplementary Table 2 for statistics.

We compared the desensitization induced by our panel of agonists in two different assays; rapid homologous desensitization taking place over 2′ of continuous agonist exposure and chronic heterologous desensitization assessed following 10, 30, 120, and 240′ of prior agonist exposure. *Rapid desensitization*: An example of the decline in VACC inhibition induced by the continuous perfusion of a MOPr agonist is shown by the exemplar trace in Figure 3A. Using this assay, we found that limited desensitization was induced by both NKTR-181 (10 and 30 μM) and oxycodone (10 μM). In contrast, DAMGO, fentanyl, or morphine induced greater desensitization than NKTR-181 (30 μM, Figure 3A, Table 1, Supplementary Table 2). *Chronic desensitization*: In this assay we used the maximal dose of a full agonist, DAMGO (1 μM), to assess MOPr-VACC inhibition following increasing pre-incubation times of NKTR-181 (30 μM), DAMGO (1 μM), fentanyl (1 μM), morphine (10 μM), and oxycodone (10 μM). We found that the full agonists DAMGO and fentanyl induced desensitization after 30, 60, 120, and 240′, with fentanyl also inducing desensitization after 10′. In contrast, NKTR-181 induced limited desensitization at 120′, but at no other timepoint (Supplementary Table 2), and neither morphine nor oxycodone induced desensitization at any stage (Figure 3B, Supplementary Table 2).

#### NKTR-181 Results in Greater Internalization Than Oxycodone

**Figure 4:**
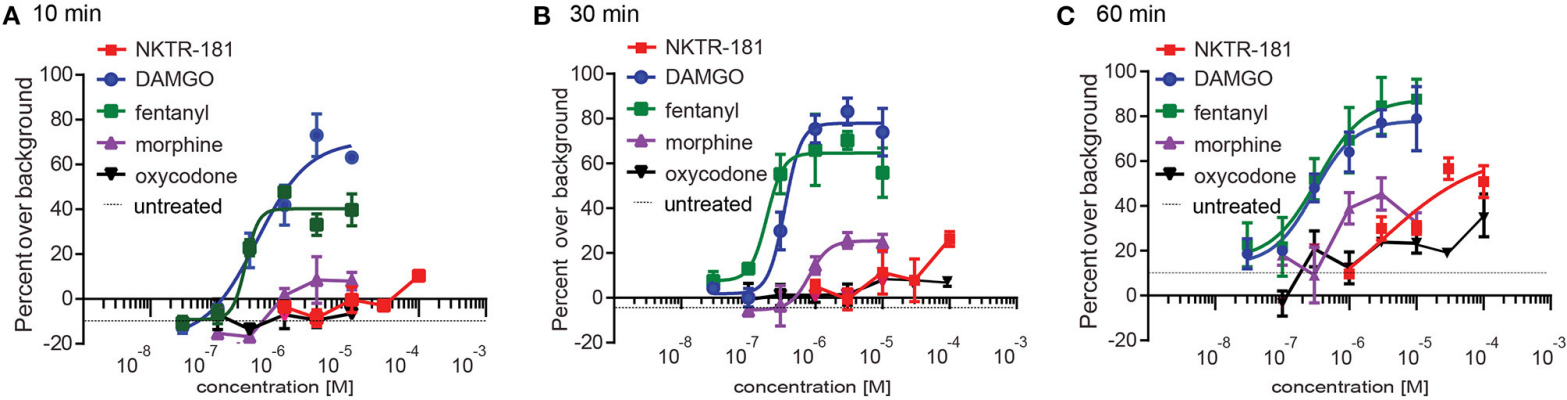
NKTR-181 induces limited internalization. Internalization was assessed in HEK cells expressing FAP-labeled MOPrs and quantified by flow cytometry. **(A)** After 10 min of agonist incubation at 37°C, DAMGO and fentanyl, but no other agonist, induced dose-dependent internalization. **(B)** After 30 min of incubation, DAMGO, fentanyl, and morphine induced dose-dependent internalization. **(C)** After 60 min of incubation, DAMGO, fentanyl, and NKTR-181 had induced dose-dependent internalization. Oxycodone did not induce dose-dependent MOPr internalization and could not be curve-fitted. The control samples indicated as a single broken black line, received vehicle at each of these timepoints. Data are shown as mean ± SEM. Refer to Supplementary Table 4 and Table 2 for statistics.

We defined the profile of MOPr internalization by NKTR-181 in comparison with oxycodone, DAMGO, and fentanyl in HEK cells. We used FAP technology to label internalized receptors (25) after increasing the doses of an agonist, 30 nM – 10 μM for DAMGO and fentanyl, 100 nM−10 μM for morphine, 100 nM−100 μM for oxycodone, and 1–100 μM for NKTR-181, after 10, 30, and 60′ of incubation at 37°C. We found that DAMGO- and fentanyl induced internalization after 10′ (Figure 4A, Table 4). By 30′, when commonly assessed MOPr agonists have induced peak levels of internalization (26), DAMGO, fentanyl, and morphine showed dose-dependent internalization profiles (Figure 4B, Table 4). By 60′, NKTR-181 induced dose-dependent internalization but this was ~10-fold less potent than DAMGO or fentanyl whose EC50 and Emax remained unchanged (Figure 4C, Table 4). Oxycodone did not result in a dose-dependent effect and could not be curve-fitted at any timepoint (Table 4).

**Table 4:** The internalization profile of NKTR-181 compared with oxycodone and other MOPr agonists after increasing lengths of agonist incubation.

**Compound**	**10 min; EC50 (nM)**	**10 min; Emax (% background)**	**30 min; EC50 (nM)**	**30 min; Emax (% background)**	**60 min; EC50 (nM)**	**60 min; Emax (% background)**
NKTR-181	–	–	–	–	2,943	65 ± 43
Oxycodone	–	–	–	–	–	–
DAMGO	391	71 ± 11	350	78 ± 6	317	72 ± 7
Fentanyl	262	40 ± 3	189	67 ± 6	266	83 ± 9
Morphine	–	–	819	25 ± 4	–	–

#### The Mechanism of Inhibition of VACCs Using Both NKTR-181 and Oxycodone Shows Differences but Also Similarities

**Figure 5:**
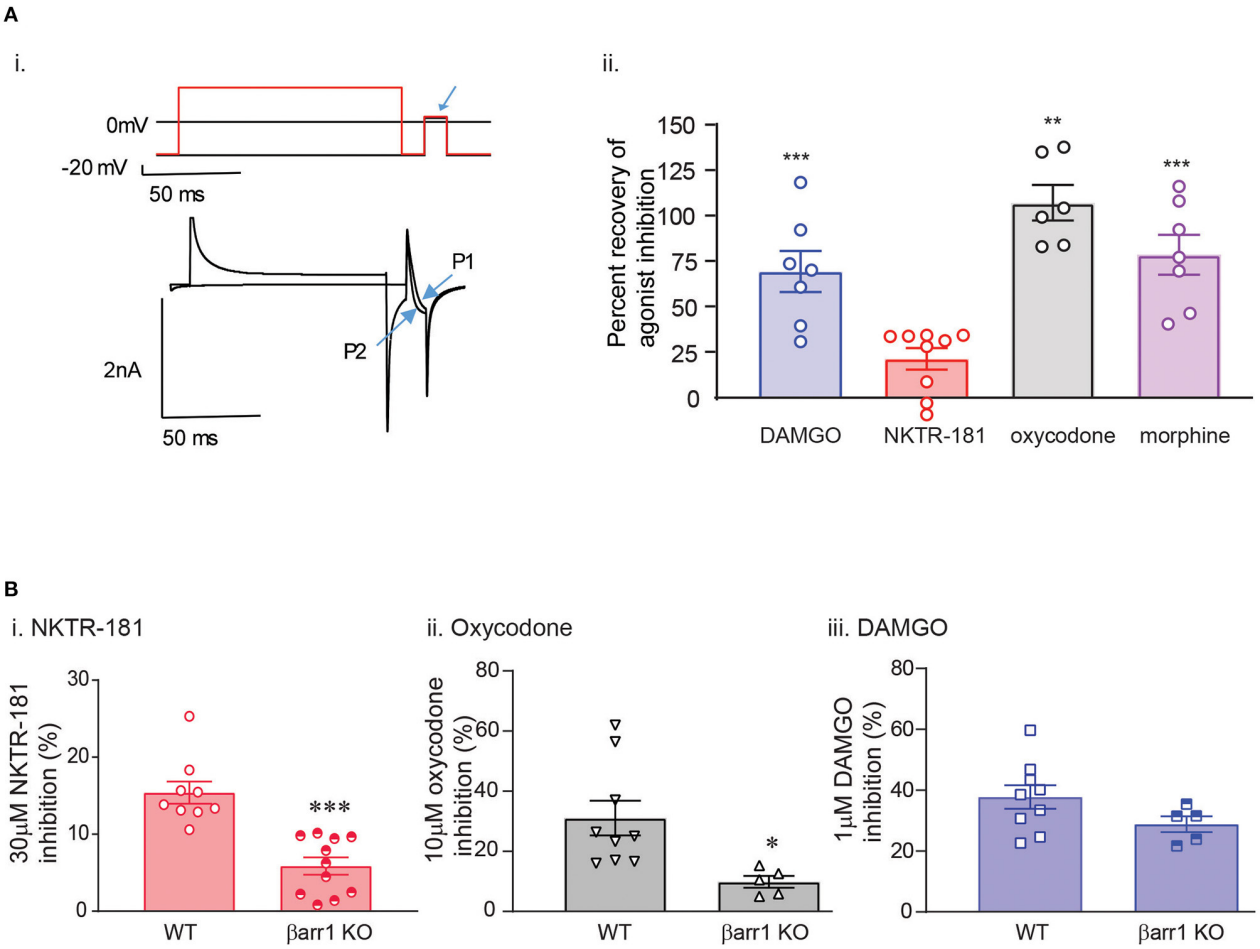
The mechanism of NKTR-181 inhibition of VACCs is both different and similar to that of oxycodone. This was assessed by voltage-clamp patch-clamp recordings in adult DRG neurons. **(A)** *Voltage-dependent inhibition*. [A(i)] Agonist-VACC inhibition was first obtained and followed by a two-pulse protocol occurring over two sweeps in the presence of the agonist. In the first sweep (shown in black), there was no high voltage pre-pulse prior to the test pulse (highlighted by the blue arrow) and in the second sweep (shown in red) a high-voltage pre-pulse precedes the test pulse. The change in current amplitude in the absence and presence of the high voltage pre-pulse (P1 and P2) was used to assess the percent recovery of agonist inhibition by the pre-pulse. [A(ii)] This showed that oxycodone, DAMGO, and morphine, but not NKTR-181, predominantly inhibit VACCs by a voltage-dependent mechanism. **, ***p < 0.01, 0.001 vs. NKTR-181. **(B)** *The role of the nonvisual arrestin isoform*, β*arr1*. Using DRGs lacking βarr1, we found that NKTR-181 [B(i)] requires this isoform for full VACC inhibition (*, **p < 0.001 vs. WT). Oxycodone [B(ii)], but not DAMGO [B(iii)] also requires βarr1 for full VACC inhibition (*p < 0.05 vs. WT). Data are shown as mean ± SEM. Refer to Supplementary Table 2 for statistics.

So as to further define the signaling profile of NKTR-181, we probed the mechanism and profile of NKTR-181-VACC inhibition in DRG neurons in several assays. (*a). Voltage-dependent VACC inhibition*: We first defined the voltage-dependent component of NKTR-181-VACC inhibition by using a high voltage pre-pulse to dissociate Gβγ subunits that inhibit VACCs in a voltage-dependent manner (16, 27). In this assay, an example of which is shown in Figure 5A(i), NKTR-181 showed less voltage-dependent inhibition than DAMGO, morphine, or oxycodone (Figure 5A(ii), Supplementary Table 2). Of note is that this assay normalizes the results to the percent inhibition obtained by each agonist, so the results are independent of the varying efficacies of these agonists. (*b). The requirement for nonvisual arrestins for full inhibitory VACC coupling by NKTR-181*: We have previously shown that opioid receptors may require βarr1 or βarr2 for full inhibitory coupling. For βarr2, this is due to an increase in basal constitutive coupling, thereby reducing the receptors available for ligand-dependent signaling (16), whereas βarr1 is required for VACC inhibition by some receptors but not for the MOPr agonists, morphine, or DAMGO (28). We therefore focused on βarr1 and using βarr1-knockout mice, we assessed VACC inhibition obtained by NKTR-181 (30 μM) in adult DRG neurons lacking this non-visual arrestin isoform. In these assays, both NKTR-181 and oxycodone, but not DAMGO, require βarr1 to obtain full VACC inhibition (Figures 5B(i)**–B**(iii), Supplementary Table 2).

### Behavioral Experiments

#### Nociception

Pain is a complex, multidimensional experience and an unpleasant, emotional component of pain (or how much the pain is bothersome and interferes with daily life) is considered more predictive of quality of life than its sensory component. We therefore assessed both the sensory (thermal withdrawal thresholds) and unpleasant tonic aversive components of the pain experience associated with an acute inflammatory nociception model. This is now considered necessary for a more thorough profile that will better inform the potential for clinical translation.

#### Oxycodone and NKTR-181 Have Anti-hyperalgesic Effects

**Figure 6:**
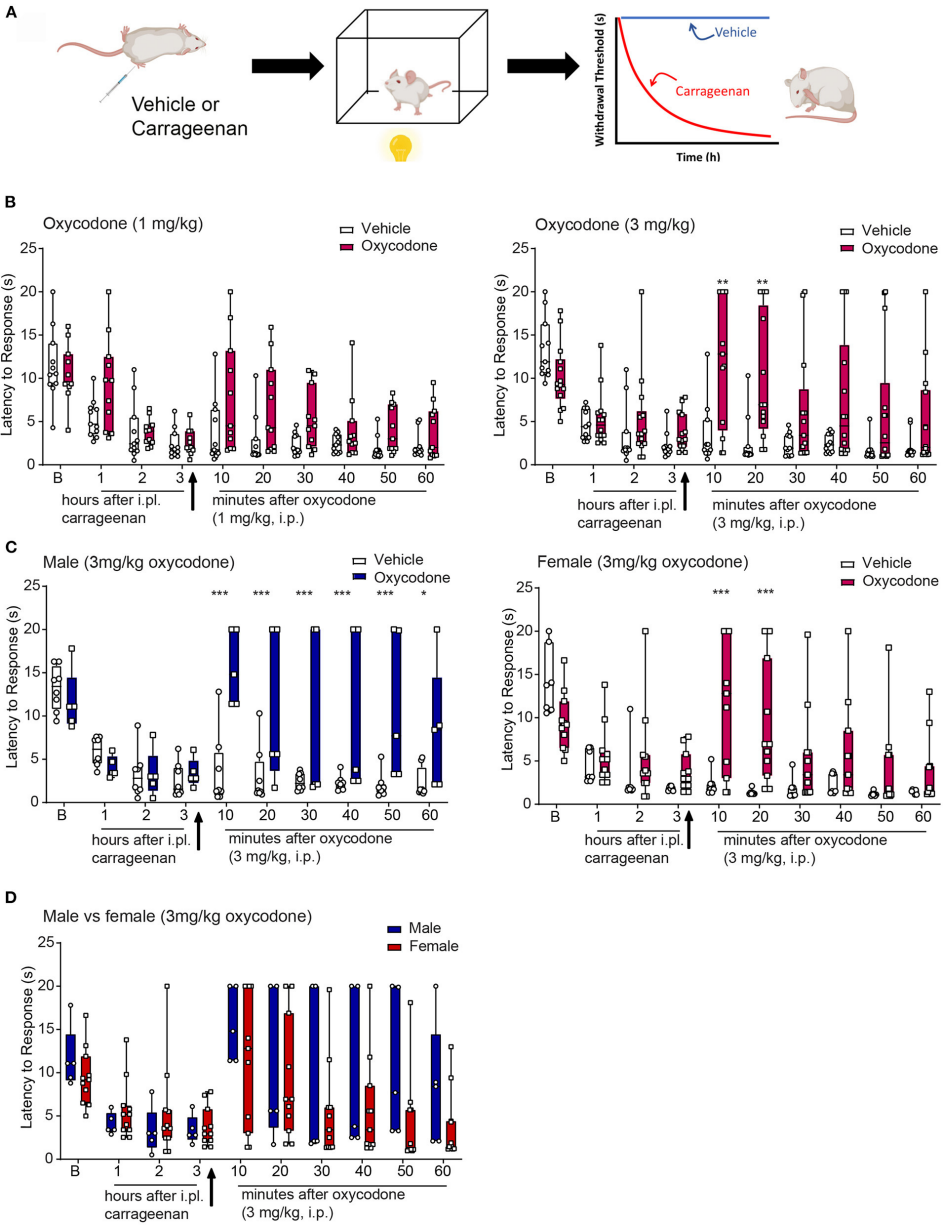
Anti-hyperalgesic effects of oxycodone in the thermal plantar test following intra-plantar (i.pl.) injection of carrageenan. **(A)** Schematic image of a protocol. Mice received an i.pl. injection of carrageenan (2%) into the left hindpaw. Baseline latencies were acquired prior to injection and mice were tested once in an h for 3 h. To evaluate the effectiveness of the opioid, oxycodone, or vehicle was injected immediately after the 3rd hour. Following drug or vehicle injection, mice were tested every 10 min for 1 h. **(B)** Oxycodone (3 mg/kg, but not 1, mg/kg, i.pl.) produced an increase in thermal withdrawal thresholds associated to vehicle. *p < 0.05, **p < 0.01, ***p < 0.001. **(C,D)** Data from the higher dose of oxycodone in panel b was divided by sex. Oxycodone produced anti-hyperalgesic effects in both males and females, with no significant differences between sexes although there was a trend that the duration of effect was longer in males. All raw data are presented as box plots with minimum to maximum points. Refer to Supplementary Table 3 for statistics.

**Figure 7:**
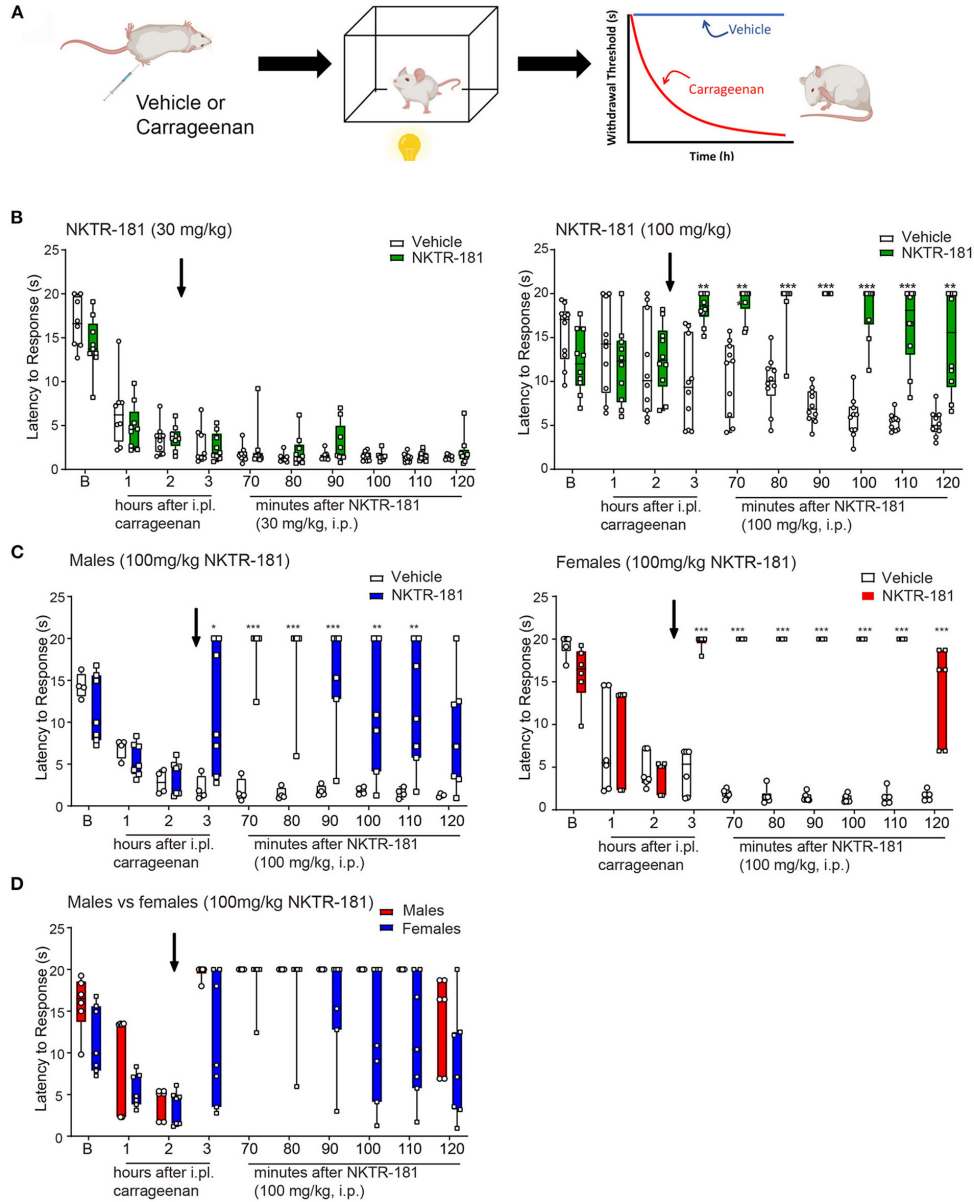
Anti-hyperalgesic effects of NKTR-181 in the thermal plantar test following an i.pl. injection of carrageenan. **(A)** Schematic image of a protocol. Mice received an i.pl. injection of carrageenan (2%) into the left hindpaw. Baseline latencies were acquired prior to injection, and mice were tested once an hour for 3 h. To evaluate effectiveness of the opioid, NKTR-181 was injected after the 2nd h due to its delayed effectiveness. Following drug or vehicle injection, mice were tested every 10 min for 1 h. **(B)** I.pl. carrageenan injection and systemic vehicle significantly reduced thermal withdrawal thresholds. NKTR-181 (100, but not 30 mg/kg, i.pl.) significantly increased thermal thresholds following i.pl. carrageenan. *p < 0.05, **p < 0.01, ***p < 0.001. **(C,D)** Data from the higher dose of NKTR-181 in panel b were divided by sex. NKTR-181 produced anti-hyperalgesic effects in both males and females, but with no effect of gender. All raw data are presented as box plots with minimum to maximum points. Refer to Supplementary Table 3 for statistics.

To determine the ability of opioid agonists to modulate inflammatory nociception, thermal withdrawal thresholds in male and female mice were determined using the Hargreaves test. To assess the anti-hyperalgesic effects, drugs were evaluated following an i.pl. injection of saline or carrageenan, respectively. Figure 6A depicts the experimental protocol of a typical hyperalgesic response displayed by the carrageenan-injected mice (29, 30). Prior to carrageenan, mice displayed thermal thresholds ranging from 5 to 20 s, with the mean of ~10–13 s for each cohort. There was no sex difference in baseline thresholds. Following injection of i.pl. saline (vehicle) threshold latencies tended to lower (Supplementary Figure 4) but there was no significant difference from baseline thresholds. In contrast, carrageenan produced a decrease in thermal withdrawal thresholds that peaked 3-h postinjection, interpreted as the development of thermal hyperalgesia (Figures 6, 7, white bars). We tested the effect of oxycodone or NKTR-181 on this hyperalgesia.

#### Oxycodone

The administration of oxycodone (3, but not 1, mg/kg i.pl.) increased withdrawal thresholds following i.pl. vehicle (Supplementary Figure 4). Similarly, 3, but not 1, mg/kg i.pl. increased withdrawal thresholds following i.pl. carrageenan (Figures 6B,D, Supplementary Table 3). No sex differences were evident in oxycodone-induced antinociception or anti-hyperalgesia, thus sexes were combined (Figure 6D).

#### NKTR-181

The administration of NKTR-181 (100, but not 30, mg/kg, i.pl., Supplementary Table 3) increased withdrawal thresholds following i.pl. vehicle (Supplementary Figure 4). Similarly, 100, but not 30, mg/kg i.pl., of NKTR-181 increased withdrawal thresholds following i.pl. carrageenan (Figure 7B, Supplementary Table 3). A sex difference was evident in NKTR-181-induced anti-hyperalgesia (Figures 7C,D). That is, NKTR-181 produced greater anti-hyperalgesic effects in male compared to female pain animals (Figures 7C,D, Supplementary Table 3).

#### Conditioned Place Preference in Pain-Naive Mice

**Figure 8:**
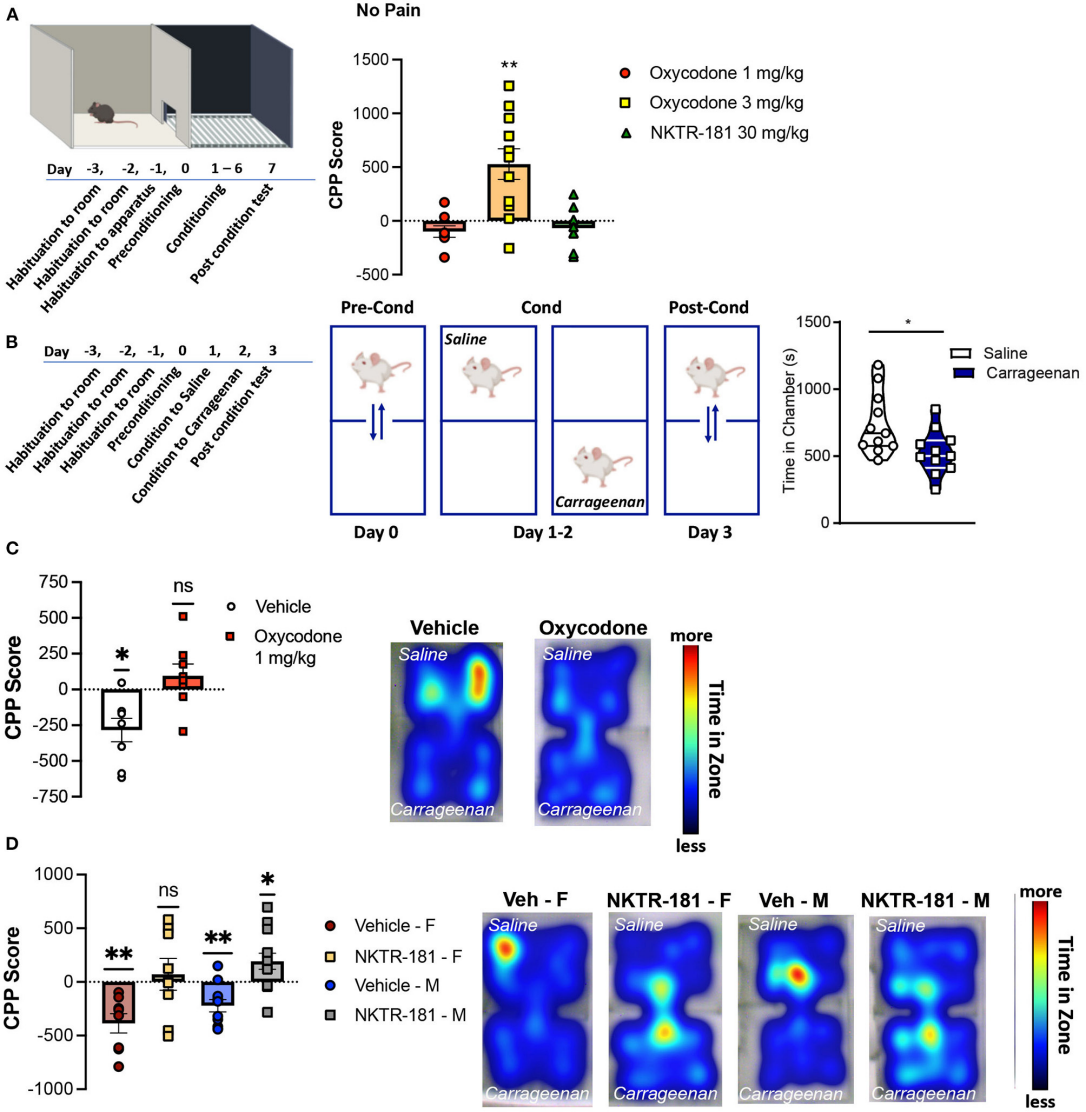
Oxycodone and NKTR-181 block pain aversion at doses that have no effect on sensory thresholds. **(A)** Time course of conditioned place preference (CPP) paradigm for oxycodone (1 and 3 mg/kg, i.pl.) and NKTR-181 (30 mg/kg, i.pl.) in pain-naïve male mice. Conditioning was carried out once a day (drug or vehicle) for 6 days and post-conditioning was in a drug-free state. Only the higher dose of oxycodone produced a place preference. **(B)** Time course and paradigm for carrageenan-induced conditioned place aversion (CPA). Mice were conditioned to either i.pl. vehicle or carrageenan (2%) beginning 1 h after injection. **(C,D)** Carrageenan-induced a place aversion in both male and female mice (left panels, *p = 0.0119). **(C)** Oxycodone (1 mg/kg, i.pl., *p* = 0.2785) blocked carrageenan-induced place aversion. Heat maps represent time in each conditioning zone. **(D)** The dose of NKTR-181 (30 mg/kg, i.pl.) that had no place preference in non-pain animals blocked carrageenan-induced place aversion. Heat maps represent time in each conditioning zone. All data are presented as CPP score. *p < 0.05, **p < 0.01. Refer to Supplementary Table 3 for statistics.

The effect of drug preference following the systemic administration of oxycodone and NKTR-181 was assessed using a CPP paradigm in mice. An unbiased counterbalanced two chamber design was used where animals spent equal time in both conditioning chambers during the habituation and pre-conditioning phases (Figure 8A). Systemic administration of 3 mg/kg i.pl. oxycodone produced a place preference in both sexes, so data were combined. However, 1 mg/kg oxycodone i.pl., the dose that did not reverse carrageenan-induced hyperalgesia, also did not produce a place preference in either male or female mice. Similarly, the use of a lower dose of NKTR-181 (30 mg/kg, i.pl.) had no effect in reversing carrageenan-induced hyperalgesia and did not produce a place preference in pain-naïve male or female mice (Figure 8A).

The time course of carrageenan-induced CPA is presented in Figure 8B. A 2-day conditioning paradigm was sufficient for carrageenan to produce a place aversion. To determine whether a non-rewarding dose of oxycodone could block carrageenan-induced place aversion, male mice were injected with oxycodone (1 mg/kg, i.pl.) immediately before either saline or carrageenan injections on both conditioning days. Similar effects were evident in female mice (data not shown). Figure 8C shows that vehicle injection had no effect on carrageenan-induced place aversion (*p* = 0.0105, *t* = 3.463, df = 7), whereas oxycodone prevented place aversion (*p* = 1.2785, *t* = 1.175, df = 7). Similar results were evident following the peripheral administration of NKTR-181 (30 mg/kg, i.pl.), where this PEGylated-opioid blocked place aversion in both male (*p* = 0.0247, *t* = 2.506, df = 12) and female (*p* = 0.6485, *t* = 0.4760, df = 7) mice (Figure 8D, Supplementary Table 3). Interestingly, NKTR-181 produced a place preference in pain, but not pain-naive mice, suggesting the occurrence of negative reinforcement (Figure 8D).

#### Intravenous Self-Administration

**Figure 9:**
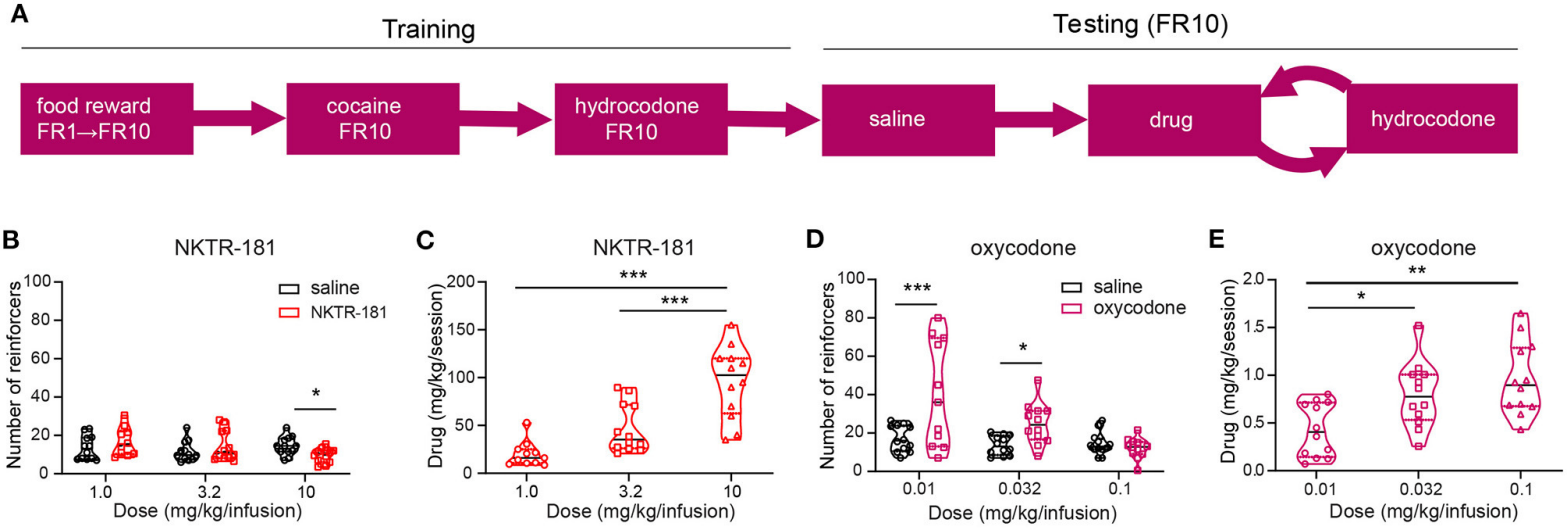
NKTR-181 generates low levels of intravenous self-administration (IVSA). **(A)** The timetable of this experiment conducted in male and female rats (*n* = 42 females and 46 males). Once stable responding had been obtained the testing schedule involved an alternating schedule of the test substance, oxocodone, NKTR-181 or saline on 3, 1 h sessions on consecutive days, and hydrocodone with each rat randomly receiving 2–3 trials of test substance. **(B)** The lower doses of NKTR-181 (1 and 3.2 mg/kg/infusion) induced the same number of reinforcers as saline and the highest dose (10 mg/kg/infusion) a lower number of reinforcers than saline. **(C)** The total amount of drug infused for each session increased by the highest (10 mg/kg) but not lower doses (1 and 3.2 mg/kg). **(D)** In contrast to NKTR-181, the low (0.01 and 0.032 mg/kg/infusion) but not high (0.1 mg/kg/infusion) dose of oxycodone-induced higher responding than saline. **(E)** When compared to the total amount of drug infused at the lowest dose, the total amount increased with each of the two higher doses. *p < 0.05, ** *p* < 0.01, and *** p < 0.001. Data are shown as violin plots. Refer to Supplementary Table 3 for statistics.

The use of an experimental design allowed a within-subject repeated measures comparison of responding to each dose of either NKTR-181 or oxycodone with saline (Figure 9A). Under the FR10 schedule, a number of reinforcers obtained by NKTR-181 did not differ from saline at the lowest doses used (1 and 3.2, mg/kg/infusion) but was reduced at the highest dose, 10 mg/kg/infusion (Figure 9B, Supplementary Table 3). Due to the increase in dose, this pattern of reinforcers obtained resulted in an increase in the total amount of drug infused over the hour-long session across doses (Figure 9C, Supplementary Table 3). Oxycodone showed a different pattern of response in that the number of reinforcers obtained were higher at the lower doses (0.01 and 0.032 mg/kg/infusion) but there was no difference at the higher dose (0.1 mg/kg/infusion, Figure 9D) in comparison with saline. This resulted in an increase in the total amount of drug infused across all doses (Figure 9E).

## Discussion

Our study demonstrates that, when compared with oxycodone, NKTR-181 has a similar but also unique pharmacology. Oxycodone was chosen as the most relevant comparator as it is a highly effective opioid in treating pain (15), it possesses high abuse and diversion rates (31), and its structure is similar to the base compound of NKTR-181, 6α-oxycodol (11). Our study confirms previous reports that NKTR-181 produces antinociceptive effects in thermal nociception assays, but our study expands this data set to show that it is also effective in blocking the tonic aversive component of nociception using nociception-induced CPA assays. Interestingly, the dose required to attenuate the emotional, affective component of nociception was lower than that required to produce antinociception. Peak effects of NKTR-181 were significantly delayed compared to oxycodone, where antinociception was not evident until 1h after injection. This delayed onset is one of the positive attributes that makes it ideally suitable as a candidate for treating OUD. This candidate would also ideally show a similar profile as other drugs used to treat OUD such as buprenorphine, in being a partial agonist with a prolonged receptor signaling profile, a slow pharmacokinetic profile, and delayed brain receptor occupancy. For this study, we have focused on the initial comparison of NKTR-181 with oxycodone to define its mechanisms of action, analgesic profile, and abuse liability. Where possible, we have also compared these agonists with well-known and studied MOPr agonists, DAMGO, fentanyl, and morphine.

### Similarities and Differences in the Signaling Profiles of NKTR-181 and Oxycodone

MOPrs can activate an array of intracellular signaling cascades but the precise signaling events or effector cascade complexes formed are ligand- or peptide-dependent (32, 33). A series of *in vitro* experiments examined these ligand-dependent differences taking care to compare each compound within each assay so as to avoid the effects of tissue, receptor expression levels, and receptor engineering.

We show that, when compared to DAMGO and fentanyl, both NKTR-181 and oxycodone are partial agonists in inhibiting VACCs and weak inducers of desensitization and internalization. However, NKTR-181 is less potent and efficacious than oxycodone at VACC inhibition, but equally efficacious at G-protein activation and β-arrestin recruitment, and induces greater internalization than oxycodone. Although neither NKTR-181 nor oxycodone are significantly biased for G-protein vs. βarr 2 recruitment, NKTR-181 shows a small but significant bias for βarr1 over Gαi, and both agonists show a bias for βarr1 over βarr2. Furthermore, NKTR-181 has a significantly higher bias for βarr1 than oxycodone. NKTR-181 also induces a slower rate of inhibition than oxycodone and predominantly inhibits VACCs in adult DRG neurons by a voltage-independent mechanism. These similarities and differences are summarized in Table 5A.

**Table 5:** Summary of the differences and similarities between NKTR-181 and oxycodone in cellular and behavioral assays.

**A. Cellular Assay**	**NKTR-181 compared with oxycodone**
*MOPr-VACC inhibition*	Both NKTR-181 and oxycodone are partial agonists, however NKTR-181 is both less potent (4 fold) and efficacious (0.5 fold) than oxycodone (Figure 1, Table 1)
*Rate of MOPr-VACC inhibition*	NKTR-181 induces a slower rate of inhibition (3 x slower) than oxycodone (Figure 1)
*Mechanism of MOPr-VACC inhibition*	NKTR-181 inhibits VACCs more by voltage-independent than voltage-dependent mechanisms. In contrast oxycodone and other MOPr agonists use predominantly voltage-dependent mechanisms (Figure 5)
*MOPr Binding*	NKTR-181 has a lower affinity for MOPr (5-10 fold) than oxycodone (Supplementary Figure 3)
*MOPr on-rate*	NKTR-181 has a slower MOPr on-rate (10 fold) than oxycodone (Supplementary Figure 3)
*Gα: βarr recruitment*	NKTR-181 is β-arr1 biased relative to Gai (Figure 2, Table 2)
*βarr2:βarr1 recruitment rate*	NKTR-181 recruits both arrestins at an equal rate but oxycodone recruits βarr2 faster than βarr1 (Figure 2, Table 3)
*βarr2:βarr1 recruitment bias*	Both agonists show a bias for βarr1 over βarr2 recruitment but NKTR-181 has a higher bias for βarr1 than 2 recruitment (Figure 2, Table 3)
*Desensitization*	Unlike the full agonists DAMGO and fentanyl, neither NKTR-181 or oxycodone induce significant rapid or chronic desensitization (Figure 3)
*Internalization*	NKTR-181 induces greater internalization than oxycodone but less than the full agonists DAMGO and fentanyl (Figure 4, Table 4)
**B. Behavioral Assay**	**NKTR-181 compared with oxycodone**
*Sensory pain*	Both NKTR-181 and oxycodone produce anti-hyperalgesia but NKTR-181 requires a 30x greater dose to do so and has a longer time-course of action (Figures 6, 7 and Supplementary Figure 4)
*Affective pain*	Both oxycodone and NKTR-181 reduce conditioned place aversion to a pain-associated environment at doses lower than required to alter sensory pain thresholds. The same differences in dose and time course as for sensory pain also occur for affective pain (Figure 8)
*Intravenous self-administration (short-access)*	Oxycodone results in an increase in lever pressing behavior with increasing doses whereas NKTR-181 does not. Oxycodone results in a graded increase in the total amount of drug infused across the 3 doses assessed (0.01, 0.032 and 0.1mg/kg). NKTR-181 results in an increase in the total amount of drug infused at the highest dose, 10mg/kg (Figure 9)

One of the marked differences between these agonists is the voltage-independent vs. voltage-dependent mechanism of inhibition. This is determined by using a high-voltage pre-pulse to dissociate channel-bound Gβγ subunits in the presence of an agonist. The resultant relief of inhibition is described as the voltage-dependent component of agonist inhibition, and the remaining inhibition termed as voltage-independent. The two mechanisms utilize different signaling pathways (34, 35). The voltage-dependent mechanism principally relies on the direct association of the Gβγ subunit with the Ca^2+^ channel, whereas the voltage-independent mechanism is generally slower and relies on second messenger intermediates such as phospholipase C (36), phosphatidylinositol 4,5-bisphosphate (37), and possible receptor–channel interactions (38). The inhibition mediated by NKTR-181 was both slower and voltage-independent, suggesting that signaling pathways different from those used by oxycodone were involved in regulating VACC inhibition.

Although it has been suggested that a signaling bias for G proteins over βarr2 results in safer analgesics that may possess less abuse liability, more recent studies do not support the hypothesis that βarr2 signaling is responsible for adverse events associated with opioids (39–42). Our studies suggest that both NKTR-181 and oxycodone are βarr1-biased relative to both Gαi and βarr2 recruitment, but NKTR-181 is significantly more βarr1-biased than oxycodone. The role of βarr1 in MOPr signaling is less well understood than that of βarr2, but our data suggest that it is required for the inhibition of VACC in response to both NKTR-181 and oxycodone, and that its role is not redundant with βarr2. Given that NKTR-181-induced regulation of VACC involves a voltage-independent mechanism that distinguishes it from oxycodone, βarr1 may play multiple roles in regulating VACC activity. βarr1 has been reported to promote the internalization of L-type Ca^2+^ channels, although this has not been examined for N-type Ca^2+^ channels, it is a potential mechanism for the slow βarr1-dependent/voltage-independent VACC inhibition observed with NKTR-181(43). Although the precise role of βarr1 remains to be determined, this low potent, partial agonist with a slower onset of cellular effect and reduced CNS entry (10, 11) possesses properties other than G-protein signaling bias that may confer lower abuse liability than oxycodone, a prescription opioid that is often abused (44).

### Both NKTR-181 and Oxycodone Attenuate the Sensory and Unpleasant Components of Nociception

Our *in vivo* experiments, summarized in Table 5B, show that NKTR-181 induces antinociception and relieves hyperalgesia in the carrageenan model of inflammatory nociception at a similar efficacy but with less potency than oxycodone. NKTR-181 is also much slower at reaching peak levels of effect, taking up to 70 min to do so compared with the 10 min, the earliest timepoint measured, for oxycodone. NKTR-181, but not oxycodone, also shows gender-specific effects. At doses shown not to alter thermal thresholds in pain-naïve mice, that were devoid of thermal anti-hyperalgesic effects in the carrageenan model, and that had no effect in CPP tests in pain-naïve mice, NKTR-181, similar to oxycodone, prevented the place aversion induced by an i.pl. injection of carrageenan.

The biggest difference between NKTR-181 and oxycodone in these models was the time course of effect, most likely due to a different kinetic profile between the drugs (10, 11, 14, 15). NKTR-181 also has a slower CNS entry compared with other opioid analgesics (11). Together these effects could account for the delay in analgesic effects relative to oxycodone, which produced maximal anti-hyperalgesic effects within 10 min of systemic administration, whereas NKTR-181 was not effective until 70-min postinjection. Previous studies reported the antinociceptive effects of NKTR-181 in acute phasic reflexive tests such as tail flick and von Frey tests (45) as well as hot plate and acetic acid writhing assays (11). Our study is the first to assess the effectiveness of NKTR-181 in a model of prolonged nociceptive hypersensitivity as well as the assessment of both anti-hyperalgesic and negative reinforcement effects associated with this model. In addition, the finding that dose differentiation was evident between the sensory and unpleasant component of the nociception experience suggests that NKTR-181 doses can be titrated to have minimal effect on sensation while still providing antinociception.

### Low Abuse Liability of NKTR-181

The gold standard for assessing drug abuse liability in rodents is an operant model of IVSA. Using the similar protocol of self-administration as previously described (11), our study shows minimal evidence of rewarding or reinforcing effects in the IVSA model. The number of reinforcers earned on a FR10 schedule showed that oxycodone (0.01–0.1 mg/kg/infusion) show a dose-dependent increase in the number of reinforcers earned similar to saline. In contrast, none of the doses of NKTR-181 elicited an increase in reinforcers earned similar to saline at any of the doses tested (1–10 mg/kg/infusion) although there was an increase in the amount of NKTR-181 obtained per session at the highest dose (10 mg/kg/infusion) suggesting some rewarding effect at this high dose. A previous study using a progressive ratio task, where with each reinforcer earned, the work effort was made exponentially harder, NKTR-181 was not found to be reinforcing at any of the doses tested to a maximum of 3.2 mg/kg/infusion, despite blood levels as high as >5,000 nmol/kg (11).

The effective dose of oxycodone and NKTR-181 in blocking the aversive component of nociception was lower than the dose required to attenuate a threshold sensory component (hyperalgesia). The self-administration studies were conducted by an intravenous drug delivery, which used lower doses than either of the nociception assessment assays. There is no general rule of thumb for comparing the systemic peripheral administration to intravenous, but it is clear that oxycodone produces a drug reinforcement at very low doses (0.01 mg/kg), whereas NKTR-181 (10 mg/kg) had a minimal effect in producing a drug reinforcement. These data suggests that NKTR-181 likely has much lower potential for producing abuse or misuse compared to oxycodone.

Another means of addressing abuse liability is to determine potential drug-induced physical dependence, which manifests as psychological and physical withdrawal (46). In a phase III clinical trial demonstrating significant analgesic efficacy with a low incidence of CNS adverse events in low back pain patients (9), withdrawal as assessed using Clinical Opiate Withdrawal Scale (COWS) demonstrated that no patients randomized to NKTR-181 reported symptoms of moderate or severe opioid withdrawal (COWS score ≥ 13), with a mild withdrawal (COWS score < 13) being reported in 9 out of 309 patients (47). Furthermore, in non-physically dependent recreational opioid users, a randomized, double-blind single-dose crossover study showed fewer and less severe subjective effects indicating a lower opioid abuse potential of NKTR-181 compared to oxycodone (10).

### Summary

NKTR-181 was advocated as an analgesic for acute pain to an FDA Advisory Committee in early 2020. However, citing concerns about the approval of new opioids during an opioid epidemic and the lack of data on liver toxicity or misuse by various delivery methods (such as nasal inhalation), this compound did not get approval from the FDA to advance clinical development. As such, its potential as an analgesic for acute pain was withdrawn from further development by Nektar Therapeutics (48, 49). Nevertheless, our studies show that there could be interest in developing this drug as a novel treatment for OUD. NKTR-181 is indeed an opioid that possesses similarities to other opioid agonists. Like other clinically useful opioid analgesics, NKTR-181 inhibits Ca^2+^ channels and recruits G proteins and arrestins following binding to MOPr. NKTR-181 is a partial agonist similar to buprenorphine and possesses several unique properties such as a slower receptor on-rate, a slower rate and mechanism of channel inhibition and a different pattern of arrestin recruitment like oxycodone. In addition, we confirm previous reports that NKTR-181 has a reduced abuse liability compared to oxycodone. Taken together, the findings that NKTR-181 has a longer half-life, slow CNS entry, partial agonist activity and low reinforcement effects, yet exhibits efficacy as an analgesic, providing evidence that this drug may be an effective abuse deterrent treatment. Further studies would be needed to examine this possibility.

## Data Availability Statement

The original contributions presented in the study are included in the article/Supplementary Material, further inquiries can be directed to the corresponding author.

## Ethics Statement

The animal study was reviewed and approved by The Office of Animal Research Oversight, UCLA.

## Author Contributions

AL, ST, and NR designed, performed, and analyzed the BRET and internalization experiments. AL, IB, and VM designed, performed and analyzed the pain behavioral experiments. AL wrote the BRET and behavioral sections and edited the manuscript. TM, LV, and JZ provided direction and discussed all experiments and edited the manuscript. KD designed and analyzed the BRET experiments, wrote sections of the paper and edited the manuscript. CC designed and analyzed the pain behavior experiments and wrote and edited all sections of the paper. WW designed, performed and analyzed the electrophysiology experiments and wrote and edited all sections of the paper.

## Conflict of Interest

KD was employed by company KiloDalton Consulting. The remaining authors declare that the research was conducted in the absence of any commercial or financial relationships that could be construed as a potential conflict of interest.

## Publisher's Note

All claims expressed in this article are solely those of the authors and do not necessarily represent those of their affiliated organizations, or those of the publisher, the editors and the reviewers. Any product that may be evaluated in this article, or claim that may be made by its manufacturer, is not guaranteed or endorsed by the publisher.
