# Supplementary material for: In vivo and in vitro Characterization of a Partial Mu Opioid Receptor Agonist, NKTR-181, Supports Future Therapeutic Development
Source: Front Pain Res (Lausanne). 2021 Aug 23;2:695962. doi: 10.3389/fpain.2021.695962 (PMC8915576; doi:10.3389/fpain.2021.695962)
Supplement: Supplementary file 1 [file Table_1.DOCX]

**STable 1: Acceptor/Donor Titrations of MOR-venus and βarr-luciferase.** HEK293 cells, transfected with luciferase-tagged βarr1 or 2 (donor) alone or co-transfected with increasing amounts of MOR-Venus (acceptor), were untreated or treated with DAMGO (10μM, NKTR181 (100μM) or Oxycodone (100μM) for 15 minutes. Net BRET vs the ratio of acceptor to donor were graphed and fitted by non-linear regression with a single site binding model. Values from untreated cells were fit with a linear regression and the slope used to constrain the non-specific binding. BRETmax (plateau) and the BRET50 (ratio at which half maximal BRET is reached) are equivalent to the Bmax and Kd values.

|  |  | DAMGO | NKTR181 | OXY |
| --- | --- | --- | --- | --- |
| βarr2 | **BRET_50_** | 0.6±0.52 | 1.4±1 | 1.35±1.2 |
|  | **BRETmax** | 0.036±.008 | 0.02±.006 | 0.021±.006 |
| βarr1 | **BRET_50_** | 1.6±0.6 | 3.3±1.7 | 3.5±2 |
|  | **BRETmax** | 0.028±0.001 | 0.03±0.01 | 0.03±0.01 |

**STable 2.** Statistical Analysis of *in vitro* Experiments.

| **Experiment** | **Figure** | **Statistics test** | **Sample Number** | **Variables** | **Main Interaction** | **Significance** | **Post-hoc** |
| --- | --- | --- | --- | --- | --- | --- | --- |
| Agonist-VACC inhibition | 1b | Sigmoidal nonlinear fit | NKTR-181; n=36, oxycodone; n=36, DAMGO; n=36, morphine; n=62, fentanyl; n=42 |  |  |  |  |
| Slope | 1cii | Unpaired students T-test | Oxycodone n=6, NKTR-181 n=14 | NA | t=3.391, df=18 | p<0.01 |  |
| Percent inhibition | 1ciii | Unpaired students T-test | Oxycodone n=6, NKTR-181 n=14 | NA | t=2.5, df=16 | p<0.05 |  |
| Dose response G-protein, β-arrestin recruitment, Gβγ dissociation | 2a-c, Table 2 | 4 parameter nonlinear fit  Hill slope=1 | G-protein: DAMGO, NKTR-181, oxycodone, n=7; morphine, fentanyl n=5; β-arrestin-2: DAMGO, NKTR-181, oxycodone, n=11; morphine, fentanyl, n=8; β-arrestin-1: DAMGO, NKTR-181, oxycodone n=9, morphine, fentanyl, n=6 | Concentration,  Drug |  |  |  |
| Emax: G-protein recruitment | 2a  Table 2 | 1-way ANOVA | DAMGO, NKTR-181, oxycodone, n=7; morphine, fentanyl n=5; | Drug | F(4,30)=16 | p<.0001 | Tukey’s posthoc test  DAMGO vs NKTR-181, oxycodone, p<.0001  NKTR-181 vs morphine, p=.04  NKTR-181, oxycodone vs fentanyl p<.0001  Morphine vs fentanyl, p=.04 |
| EC50: G-protein recruitment | 2a  Table 2 | 1-way ANOVA | DAMGO, NKTR-181, oxycodone y, n=7; morphine, fentanyl n=5; | Drug |  |  | Tukey’s posthoc test  DAMGO vs NKTR-181, oxycodone, p<.0001  NKTR-181, oxycodone vs fentanyl p=.02 |
| Emax: β-arrestin-2 recruitment | 2b  Table 2 | 1-way ANOVA | DAMGO, NKTR-181, oxycodone y, n=11; morphine, fentanyl, n=8; | Drug | F (4, 46) = 46.6 | p<.0001 | Tukey’s posthoc test  DAMGO vs NKTR-181, p=.04  NKTR-181 vs fentanyl, p<.0001  Oxycodone vs fentanyl, p=.0004  Morphine vs fentanyl, p=.0095 |
| EC50: β-arrestin-2 recruitment | 2b  Table 2 | 1-way ANOVA | DAMGO, NKTR-181, oxycodone, n=11; morphine, fentanyl, n=8; | Drug | F (4, 46) = 8.1 | p<.0001 | Tukey’s posthoc test  DAMGO vs NKTR-181, oxycodone, morphine, p<.0001  DAMGO vs fentanyl, p=.0002  NKTR-181 vs fentanyl, p=0002  Fentanyl vs oxycodone, morphine, p<.0001 |
| Emax: β-arrestin-1 recruitment | 2c  Table 2 | 1-way ANOVA | DAMGO, NKTR-181, oxycodone; n=9, morphine, fentanyl, n=6 | Drug | F (4,41) =20.9 | P<.0001 | Tukey’s posthoc test  DAMGO vs NKTR-181, p=.0064  DAMGO vs oxycodone, morphine, p<.0001  NKTR-181 vs fentanyl, p=.0002  Oxycodone vs fentanyl, p=.0017  Morphine vs fentanyl, p<.0001 |
| EC50: β-arrestin1 recruitment | 2c  Table 2 | 1-way ANOVA | DAMGO, NKTR-181, oxycodone; n=9, morphine, fentanyl, n=6 | Drug | F (4,41) =29.8 | P<.0001 | Tukey’s posthoc test  DAMGO vs NKTR-181, p=.04  DAMGO vs oxycodone, p=.028  DAMGO vs morphine, p=.013  DAMGO, NKTR-181, oxycodone, morphine vs fentanyl, p<.0001 |
| Kinetics of β-arrestin-2 recruitment | 2d | 1 phase decay, Yo=0 | n=9 | Time |  |  |  |
| Kinetics of β-arrestin1 recruitment | 2e | 1 phase decay, Yo=0 | n=8 | Time |  |  |  |
| β-arrestin-1 and 2 recruitment rate  T_1/2_ to plateau | 2f | 2 way ANOVA | Barr2 , n=8  Barr1, n=6 | Drug | F(1,30)=8.8 | P<.0001 | Sidak’s posthoc test  Oxycodone barr2<barr1, p<.001  NKTR-181 barr1 vs barr2, p=.055 (NS)  Tukey’s posthoc test  barr2: DMG < NKTR-181, p=.001  barr1: DMG < NKTR-181, p=.0001  DMG<oxycodone, p<.0001, NKTR-181<oxycodone, p=.04 |
| Transduction Constant G-protein | Table 2 | 2 way ANOVA | DMG, NKTR-181, oxycodone, n=7, morphine, fentanyl n=5; | Drug | F (3,72)=29.2 | P<.0001 | Tukey’s posthoc test  NS diff between drugs |
| Transduction Constant β-arrestin-2 | Table 2 | 2 way ANOVA | DMG, NKTR-181, oxycodone n=11, morph, fent, n=9 | Drug | F (3,72)=29.2 | P<.0001 | Tukey’s posthoc test  NKTR-181 vs morph, p=.001  NKTR-181 vs fentanyl, p=.04  Oxycodone vs fentanyl, p=.001  Morphine vs fentanyl, p<.0001 |
| Transduction Constant β-arrestin-1 | Table 2 | 2 way ANOVA | DMG, NKTR-181, oxycodone n=10, morph, fent, n=8 | Drug | F (3,72)=29.2 | P<.0001 | Tukey’s posthoc test  NKTR-181 vs Oxycodone, p=.008  NKTR-181, oxycodone, fentanyl vs morphine, p<.0001 |
| Signaling bias G-protein vs β-arrestin | 2g, h  Table 3 | 2 way ANOVA | DMG, NKTR-181, oxycodone n=7, morph, fent, n=5 | Drug | F(2,49)=8.1 | P<.0001 | Tukey’s posthoc test  G-protein bias vs b-arr2: oxy>DAMGO, p=0.012; morphine >DMG, p<.0001, morph > NKTR-181 p=.04, morphine > oxycodone, p=.03  G-protein vs barr1, morphine>DMG, NKTR-181, oxycodone, fentanyl, p<.0001  Barrestin-1 bias vs G-protein: NKTR>DAMGO, oxy, morphine, p<.0006 |
| Signaling bias β-arrestin-1 vs β-arrestin-2 | 2g, h  Table 3 | 1 way ANOVA  Unpaired student t-test | DMG, NKTR-181, oxycodone n=11, morph, fent, n=9 | Drug | F(2,18)=17.92  (ANOVA) | P<.0001 | Tukey posthoc test  b-arr-1 bias vs b-arr-2: NKTR>DAMGO, oxy, morphine, fentanyl p<.004  unpaired t-test (two-tailed)  NKTR-181>DAMGO p=.0001  NKTR-181>oxy p=.0002  Oxy>DAMGO, p=.007 |
| Rapid desensitization | 3a | 1-way ANOVA | NKTR-181 10uM; n=6, 30uM, n=6, oxycodone; n=6, DAMGO; n=6 fentanyl n=6, morphine; n=6 | Drug | F_7,39_ = 5.73, | p<0.0001 |  |
| Chronic Desensitization; NKTR-181 | 3b | 1-way ANOVA, Dunnetts post-hoc | Untreated; n=9, 10'; n=5, 30'; n=6, 60'; n=7, 120' n=6, 240; n=9 | Time | F (5, 36) = 2.478 | p<0.05 | untreated vs 120'; p<0.05 |
| Chronic Desensitization; DAMGO | 3b | 1-way ANOVA, Dunnetts post-hoc | Untreated; n=9, 10'; n=6, 30';n=6, 60'; n=6, 120' n=6, 240; n=7 | Time | F (5, 34) = 12.73 | p<0.0001 | untreated vs 30; p<0.0001 untreated vs 60; p<0.01 untreated vs 120; p<0.001 untreated vs 240; p<0.0001 |
| Chronic Desensitization; Fentanyl | 3b | 1-way ANOVA, Dunnetts post-hoc | Untreated; n=9, 10'; n=6, 30'; n=6, 60'; n=6, 120' n=6, 240; n=6 | Time | F (5, 33) = 17.44 | p<0.0001 | untreated vs 10; p<0.0001 untreated vs 30; p<0.0001 untreated vs 60; p<0.0001 untreated vs 120; p<0.0001 untreated vs 240; p<0.0001 |
| Chronic Desensitization; Morphine | 3b | 1-way ANOVA, Dunnetts post-hoc | Untreated; n=9, 10'; n=6, 30'; n=6, 60'; n=6, 120' n=6, 240; n=6 | Time | F (5, 33) = 2.428 | p=0.0558 | untreated vs 60; p<0.05 untreated vs 240; p<0.05 |
| Chronic Desensitization; Oxycodone | 3b | 1-way ANOVA, Dunnetts post-hoc | Untreated; n=9, 10'; n=6, 30'; n=6, 60'; n=6, 120' n=6, 240; n=6 | Time | F (5, 33) = 1.569 | p=0.1961 |  |
| Internalization; 10 min | 4a, Table 4 | Sigmoidal nonlinear fit | NKTR-181; n=24, oxycodone; n=21, DAMGO; n=25, morphine; n=20, fentanyl; n=20 |  |  |  |  |
| Internalization; 30 min | 4b, Table 4 | Sigmoidal nonlinear fit | NKTR-181; n=24, oxycodone; n=22, DAMGO; n=24, morphine; n=20, fentanyl; n=24 |  |  |  |  |
| Internalization; 60 min | 4c, Table 4 | Sigmoidal nonlinear fit | NKTR-181; n=22, oxycodone; n=17, DAMGO; n=23, morphine; n=19, fentanyl; n=23 |  |  |  |  |
| Voltage-dependent VACC inhibition | 5a | 1-way ANOVA, Tukey's post-hoc | NKTR-181; n=10, oxycodone; n=6, DAMGO; n=7, morphine; n=7 | Drug | F (4, 29) = 12.24 | p<0.0001 | NKTR-181 vs DAMGO; p<0.01 NKTR-181 vs oxycodone; p<0.001 NKTR-181 vs oxycodone; p<0.001 |
| Arrestin-dependent inhibition; NKTR-181 | 5bi | 1-way ANOVA, Tukey's post-hoc | WT; n=9, Barr1 KO; n=11 | Genotype | F (2, 29) = 8.99 | p<0.001 | WT vs Barr1 KO: p<0.001 |
| Arrestin-dependent inhibition; oxycodone | 5bii | unpaired student's T-test | WT; n=9, Barr1 KO; n=5 | Genotype | t=2.651 df=12 | p<0.05 |  |
| Arrestin-dependent inhibition; DAMGO | 5biii | unpaired student's T-test | WT; n=9, Barr1 KO; n=5 | Genotype | t=1.602 df=12 | NS |  |

STable 3. Statistical Analysis for Behavior Experiments

| Assay | Figure | Drug | Pain | n | Gender | Time | ANOVA | Interaction/effect | t-test |
| --- | --- | --- | --- | --- | --- | --- | --- | --- | --- |
| Hargreaves | S1 | Oxycodone  (3 mg/kg) | Vehicle | 14-16 | Vehicle; 7M, 7F  Oxycodone; 8M, 8F | F_4,108_=3.566, **p=0.0097 | F_1,28_=9.110, **p=0.0054 | F_9,252_=3.593, ***p=0.0003 |  |
| Hargreaves | 6b | Oxycodone  (3 mg/kg) | Carrageenan | 11-14 | Vehicle; 6M, 5F  Oxycodone; 5M, 11F | F_3.3,74.4_=11.22, ***p<0.0001 | F_1,23_=8.770, **p=0.0070 | F_9,204_=5.075, ***p<0.0001 |  |
| Hargreaves | 6d | Oxycodone  (3 mg/kg) | Carrageenan | 5-11 | Male vs Female | F_9,126_=6.582,  ***P<0.0001 | F_1,14_=1.294,  p=0.2743 | F_9,126_=1.410, p=0.1906 |  |
| Hargreaves | S1 | NKTR-181  (30 mg/kg) | Vehicle | 10-12 | Vehicle; 5M, 5F  NKTR-181; 6M, 6F | F_2.913, 58.27_ = 13.96  ***p<0.001 | F_1,20_=0.8659, p=0.3632 | F_9,180_=1.931, p=0.0501 |  |
| Hargreaves | 7b | NKTR-181  (30 mg/kg) | Carrageenan | 8-9 | Vehicle; 4M, 4F  NKTR-181; 4M, 5F | F_3.478, 52.16_ = 96.73  ***p<0.0001 | F_1,15_=0.0216, p=0.8852 | F_9,135_ = 1.781  p=0.0772 |  |
| Hargreaves | S1 | NKTR-181  (100 mg/kg) | Vehicle | 10 | Vehicle; 5M, 5F  NKTR-181; 5M, 5F | F_5.4,98.1_=4.521, ***p=0.0007 | F_1,18_=77.34, ***p<0.0001 | F_9,162_=15.88, ***p<0.0001 |  |
| Hargreaves | 7b | NKTR-181  (100 mg/kg) | Carrageenan | 8-10 | Vehicle; 4M, 6F  NKTR-181; 7M, 6F | F_2.3,36.9_=16.13, ***p<0.0001 | F_1,16_=51.17, ***p<0.0001 | F_9,142_=26.87, ***p<0.0001 |  |
| Hargreaves | 7d | NKTR-181  (100 mg/kg) | Carrageenan | 6-7 | Male vs Female | F_2.52,27.63_=22.28, ***p<0.0001 | F_1,11_=8.936, *p=0.0123 | F_9,99_=1.771, p=0.0832 |  |
| CPP | 8a left panel | Oxycodone  (1 mg/kg) | na | 8 | 8M |  |  |  | t_7_=1.877, p=0.1026 |
| CPP | 8a left panel | Oxycodone  (3 mg/kg) | na | 8 | 8M |  |  |  | t_7_=3.695, **p=0.004 |
| CPP | 8a right panel | NKTR-181  (30 mg/kg) | na | 8 | 8M |  |  |  | t_7_=0.9587, p=0.3697 |
| CPA | 8b | Vehicle | Carrageenan | 11 | 11M |  |  |  | t_10_=3.065, *p=0.0119 |
| CPA | 8c left panel | Vehicle | Carrageenan | 8 | 8M |  |  |  | t_7_=3.463, *p=0.0105 |
| CPA | 8c right panel | Oxycodone  (1 mg/kg) | Carrageenan | 8 | 8M |  |  |  | t_7_=1.175, p=0.2785 |
| CPA | 8d top left panel | Vehicle | Carrageenan | 8 | 8F |  |  |  | t_7_=4.284, **p=0.0036 |
| CPA | 8d top right panel | NKTR-181 (30 mg/kg) | Carrageenan | 8 | 8F |  |  |  | t_7_=0.4760, p=0.6485 |
| CPA | 8d bottom left panel | Vehicle | Carrageenan | 11 | 11M |  |  |  | t_10_=3.832, **p=0.0033 |
| CPA | 8d bottom right panel | NKTR-181 (30 mg/kg) | Carrageenan | 13 | 13M |  |  |  | t_12_=2.566, *p=0.0247 |
| IVSA | 9a | NKTR-181 (1, 3.2, 10/infusion) |  | 12 | 6M, 6F  (2M outliers) |  | F (2, 31) = 6.066, p=0.006 | 10; p<0.05 vs saline |  |
| IVSA | 9b | NKTR-181 (1, 3.2, 10 mg/kg) |  | 12 | 6M, 6F  (1M outlier) |  | F (2, 32) = 22.18, p<0.0001 | 1 vs 10; p<0.001 3.2 vs 10; p<0.01 |  |
| IVSA | 8c | Oxycodone (0.01. 0.032. 0.1 mg/kg/infusion) |  | 12 | 6M, 6F  (1M outlier) |  | F (2, 32) = 8.515, p=0.001 | 0.0032; p=0.0025 vs saline 0.01; p<0.0001 vs saline |  |
| IVSA | 9d | Oxycodone (0.01. 0.032. 0.1 mg/kg) |  | 12 | 6M, 6F |  | F (2, 33) = 7.751, p=0.0017 | 0.01 vs 0.032; p<0.05 0.01 vs 0.1; p<0.01 |  |

**STable 4. Affinity and kinetics of binding to MOR** were determined in competitive radioligand binding assays (conducted by Eurofins Panlabs Discovery Services Taiwan, Ltd), using membranes prepared from CHO-K1 cells heterologously expressing recombinant human receptor. Briefly, membranes were incubated with increasing concentrations of test compounds (each concentration in duplicate) in the presence of 0.5 nM [^3^H]naloxone in assay buffer (50 mM Tris-HCl, pH 7.4) for 30 min (equilibrium mode) or 0.5 – 90 min (kinetic mode) at 25 °C. Nonspecific binding was measured in the presence of 10 μM naloxone. Incubations were terminated by rapid vacuum filtration through GF/B filter plates (Perkin Elmer) to separate bound and free [^3^H]naloxone. Filter-bound radioactivity was quantified using a liquid scintillation counter. Experimental data were analyzed using GraphPad Prism 5.04 (GraphPad Software, Inc., San Diego, CA). IC50 values were converted to inhibitor constant values (K_i_) using published equations (Cheng & Prusoff, 1973) K_d_ values of [^3^H]naloxone were obtained from saturation binding experiments (data not shown). The binding kinetics of unlabeled ligands in competition with the radioligand were quantified using the equations of Motulsky and Mahan, (Motulsky & Mahan, 1984). The equilibrium dissociation constant K_d_ was calculated as K_d_ = k_off_/k_on_. The dissociation half-life (t_1/2_) was calculated as t_1/2_ = 0.69/k_off_.

| Ligand | k_on_ (M^-1^min^-1^) | k_off_ (min^-1^) | K_d_ (nM) | K_i_ (nM) | t_1/2_ (min) |
| --- | --- | --- | --- | --- | --- |
| NKTR181 | 5.45 x 10^5^ | 4.43 x 10^-1^ | 813 | 764 | 1.56 |
| Oxycodone | 8.68 x 10^6^ | 5.54 x 10^-1^ | 63.8 | 47.6 | 1.25 |

**References**

Cheng Y, & Prusoff WH (1973). Relationship between the inhibition constant (K1) and the concentration of inhibitor which causes 50 per cent inhibition (I50) of an enzymatic reaction. Biochem Pharmacol 22**:** 3099-3108.

Motulsky HJ, & Mahan LC (1984). The kinetics of competitive radioligand binding predicted by the law of mass action. Mol Pharmacol 25**:** 1-9.
