# Supplementary figures and images for: In vivo and in vitro Characterization of a Partial Mu Opioid Receptor Agonist, NKTR-181, Supports Future Therapeutic Development
Source: Front Pain Res (Lausanne). 2021 Aug 23;2:695962. doi: 10.3389/fpain.2021.695962 (PMC8915576; doi:10.3389/fpain.2021.695962)

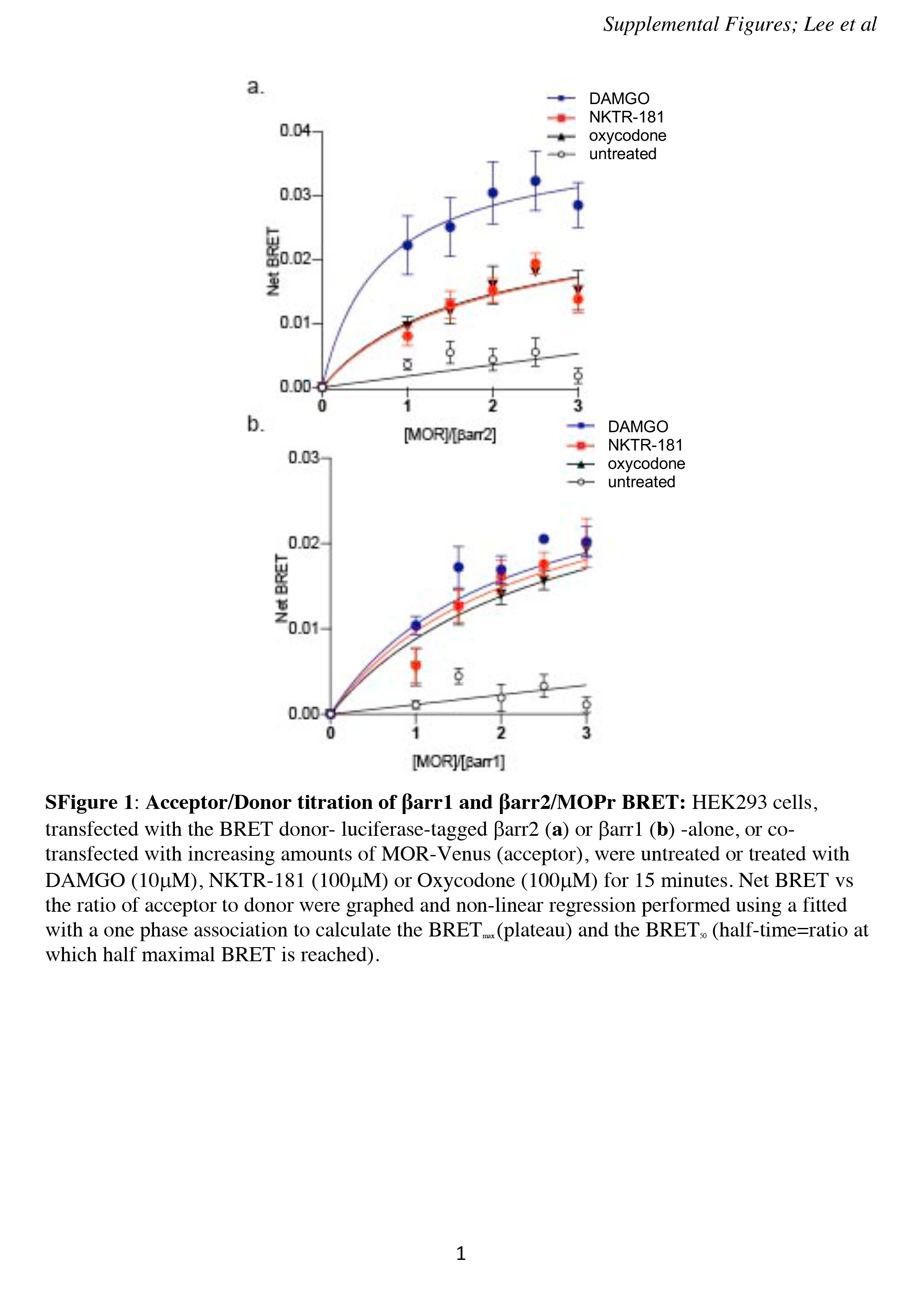

Supplement: Supplementary file 2 [file Image_1.JPEG]

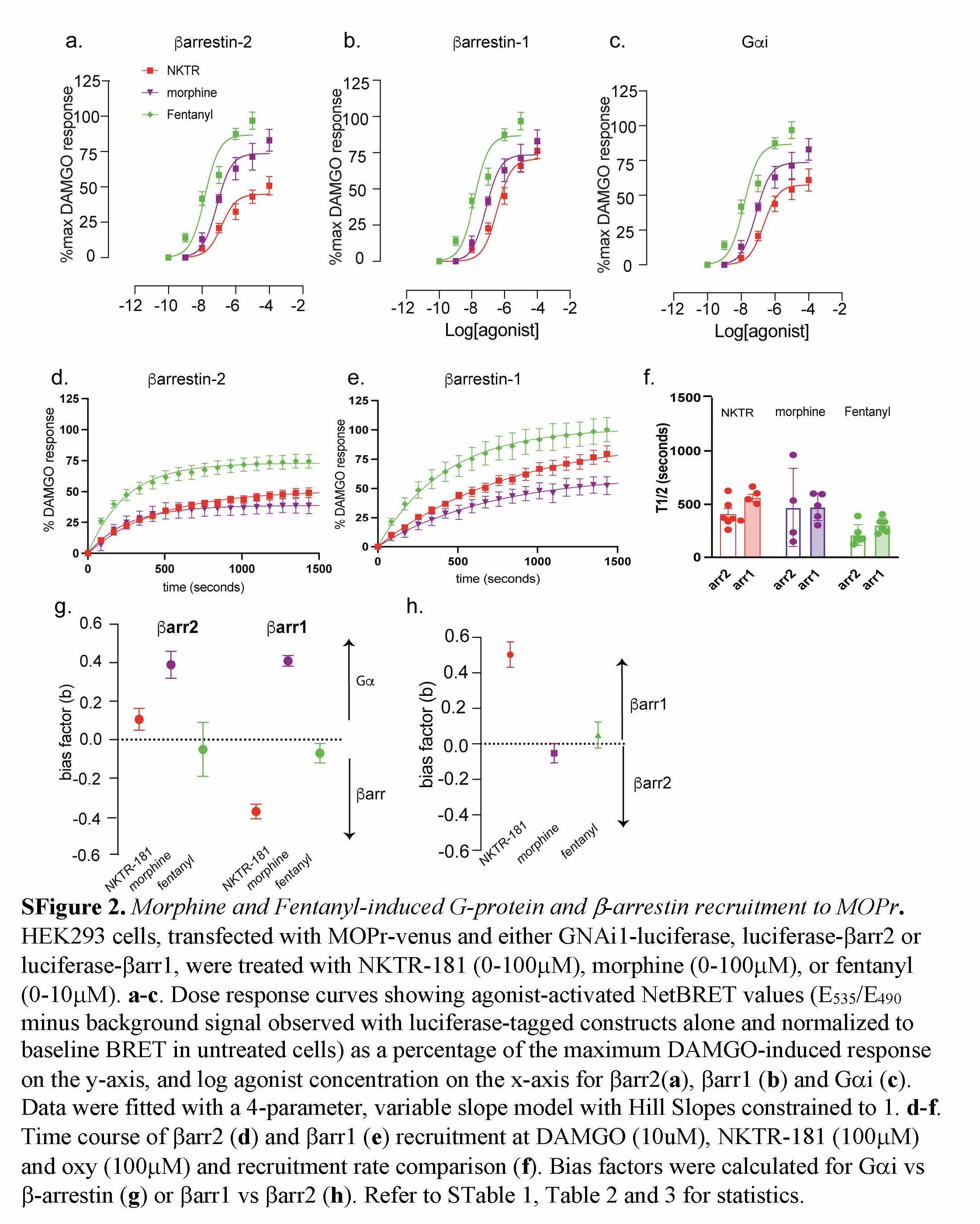

Supplement: Supplementary file 3 [file Image_2.JPEG]

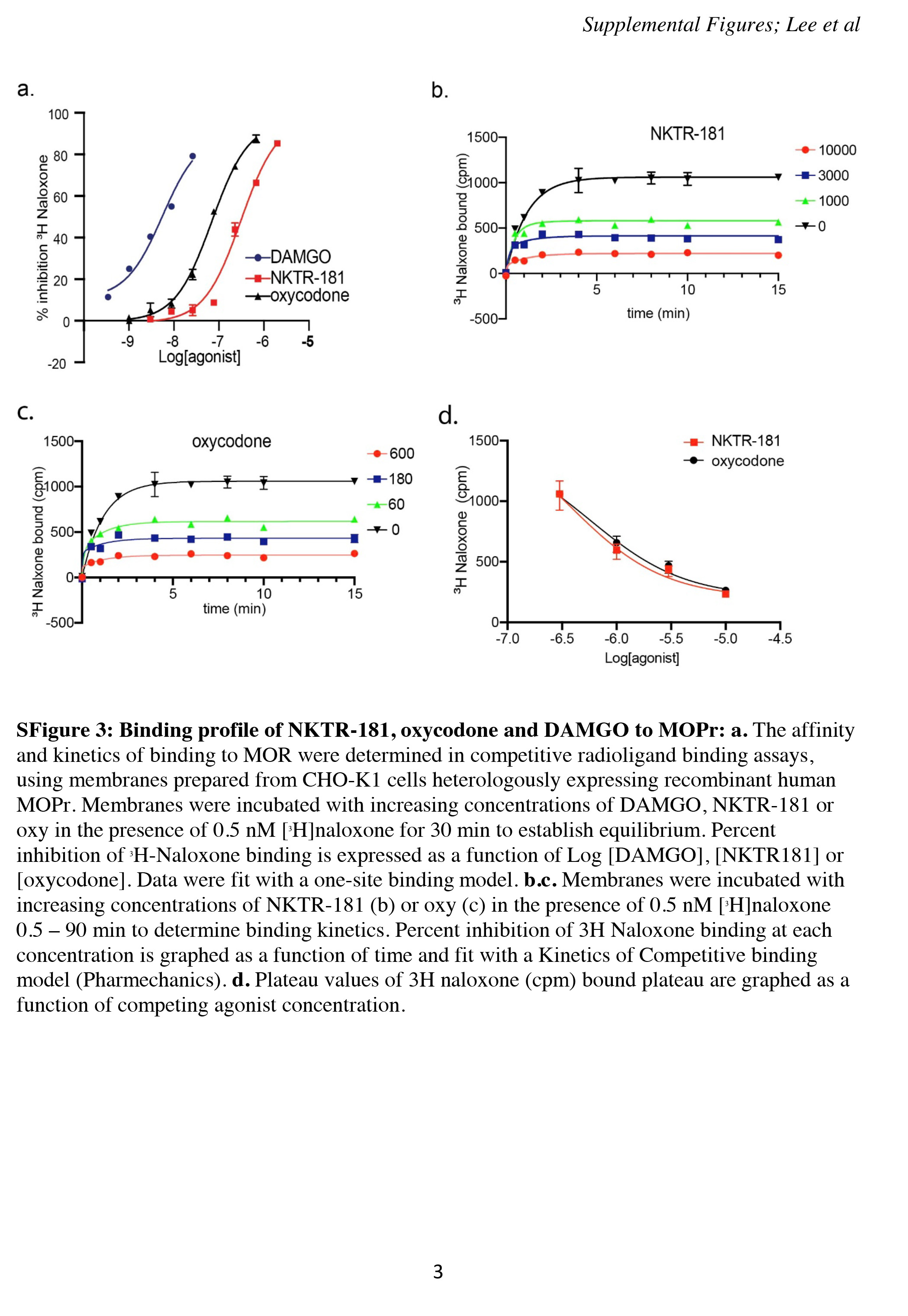

Supplement: Supplementary file 4 [file Image_3.JPEG]

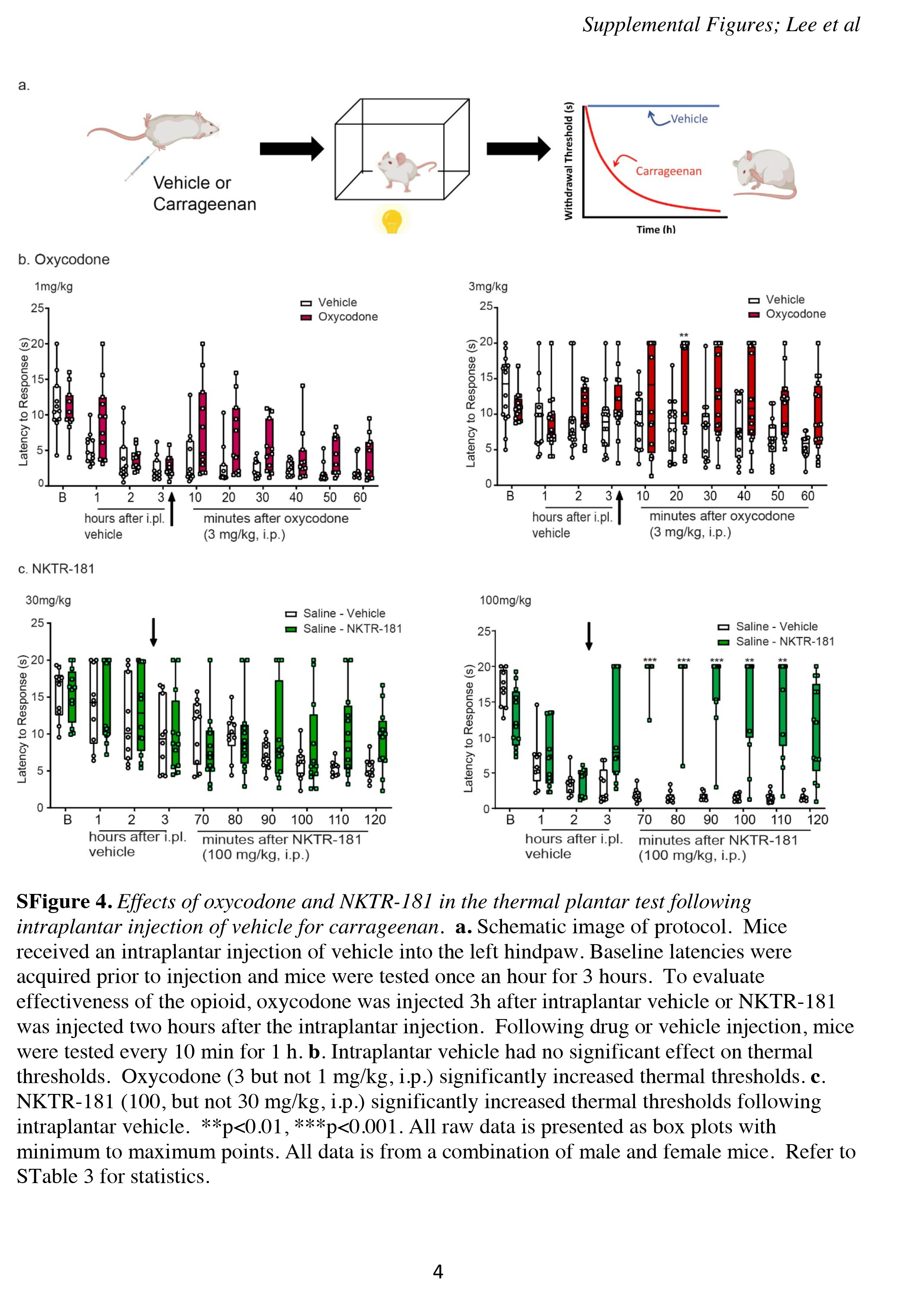

Supplement: Supplementary file 5 [file Image_4.JPEG]
